# Hobby engagement and all-cause and cause-specific mortality risk among people aged 50 years and older in 19 countries

**DOI:** 10.7189/jogh.15.04181

**Published:** 2025-07-01

**Authors:** Yujia Guo, Fan Yang

**Affiliations:** 1 School of Social Development and Public Policy, Fudan University, Shanghai, China; 2 School of Public Health, Fudan University, Shanghai, China; 3 NHC Key Lab of Health Technology Assessment, Fudan University, Shanghai, China

## Abstract

**Background:**

Global population ageing necessitates identifying modifiable factors for healthy longevity. Hobby engagement emerges as a promising yet unexplored factor; evidence of its protective effects on all-cause and cause-specific mortality risk has never been examined at a multinational level.

**Methods:**

We investigated hobby engagement and mortality risk among 79 464 adults aged ≥50 across 19 countries using harmonised longitudinal ageing cohorts. Cox proportional hazards models examined associations between hobby engagement and all-cause mortality. Competing risk models assessed cause-specific mortality. Marginal structural models evaluated the impact of change patterns in hobby engagement over time.

**Results:**

Hobby engagement was associated with a 29% reduction in all-cause mortality risk across 19 countries (pooled hazard ratio (HR) = 0.71; 95% confidence interval (CI) = 0.67, 0.75). Population attributable fractions ranged from 3.03% (in Denmark) to 23.56% (in China), with potential gains in life expectancy from 0.06 years (in China) to 1.02 years (in Sweden) over five years. Region-specific protective effects emerged: reduced mortality from endocrine/metabolic (subhazard ratio (SHR) = 0.31) and neurological conditions (SHR = 0.51) in the USA; cardiovascular mortality (SHR = 0.56) in England; and heart attack (SHR = 0.77), stroke (SHR = 0.62), other cardiovascular-related illnesses (SHR = 0.82), and respiratory disease (SHR = 0.68) in Europe. Hobby engagement patterns varied dramatically across countries, from predominant non-engagement in China (65.1%) to high sustained engagement in Northern Europe (>90%). Both initiating (pooled HR = 0.62) and sustaining (pooled HR = 0.45) hobby engagement were associated with a reduction in mortality risk compared to sustained non-engagement, while cessation eliminated these protective associations (pooled HR = 0.96; 95% CI = 0.88, 1.04). Benefits were more pronounced among adults aged ≥65 and married individuals.

**Conclusions:**

Hobby engagement is a potentially universal, modifiable factor for promoting global healthy longevity. Public health strategies prioritising initiating and maintaining hobby engagement could yield substantial survival benefits, particularly in countries with predominant non-engagement patterns and high preventable mortality.

Global population ageing demands innovative strategies to promote healthy longevity [1]. Hobbies – characterised by intentional engagement and enjoyment [2] in chosen activities like arts, crafts, reading, games, sports, gardening, volunteering, and club participation [1,3] – they differ fundamentally from general leisure activities in terms of structured engagement and personal investment. This distinction, combined with their modifiability and accessibility, positions hobbies as particularly promising targets for health promotion [4,5]. Evidence suggests that hobbies have diverse health benefits, including enhanced mental well-being [1], reduced risk of cardiovascular diseases [3], frailty [6], and dementia [7,8], particularly among older adults.

However, whether hobby engagement can confer survival benefits on a global scale remains a critical yet unexplored question for public health policy. Previous studies examining hobby engagement and mortality risk have been limited to specific hobby types within a single country [9–15]. Current research is constrained by its focus on particular hobbies within single countries, lacking evidence across nations with different participation patterns. Moreover, evidence regarding hobby engagement's relationship with cause-specific mortality (*e.g.* mortality from endocrine/metabolic, cancer, and respiratory disease) remains lacking. This knowledge gap hinders the development of global strategies to promote healthy ageing through hobby engagement. Furthermore, existing research has relied on static measurements of hobby engagement, failing to capture its dynamic nature among older adults [16,17].

To address these limitations, we analyse harmonised data from four nationally representative, longitudinal ageing studies across 19 countries in China, the USA, England, and Europe. This approach enables multi-national examination of all-cause and cause-specific mortality risks associated with hobby engagement, while exploring the impact of changing engagement patterns over time.

## METHODS

### Study design and participants

We used harmonised longitudinal ageing cohorts data across 19 countries from four nationally representative longitudinal health surveys of community-dwelling older adults (≥50 years): the US Health and Retirement Study (HRS) [18], English Longitudinal Survey of Ageing (ELSA) [19], Survey of Health, Ageing and Retirement in Europe (SHARE: Austria, Belgium, Czech Republic, Denmark, Estonia, France, Germany, Hungary, Italy, Netherlands, Poland, Portugal, Slovenia, Spain, Sweden, and Switzerland) [20], and China Health and Retirement Longitudinal Study (CHARLS) [21], which were designed using similar survey protocols to enable cross-regional comparisons (Methods S1 and Table S1 in the **Online Supplementary Document**). We followed the Strengthening the Reporting of Observational Studies in Epidemiology (STROBE) reporting guidelines for prospective cohort studies to report our findings.

We included participants aged ≥50, excluding those with missing information on hobby engagement at baseline, those who were lost to follow-up after the first survey, and those with missing values for baseline covariates (Figures S1–4 in the **Online Supplementary Document**).

### Assessment of hobby engagement

The measurement of hobby engagement and response categories varied slightly across countries (Table S2 in the **Online Supplementary Document**), reflecting cultural differences among the 19 countries studied. Following previous research [1], we created a binary hobby engagement indicator (yes/no) for each country to ensure data comparability.

To further dynamically assess hobby engagement over time, we draw on similar research [22] to construct four change patterns of hobby engagement using two exposure time points during the follow-up period. These patterns were defined using a moving window of two consecutive interviews immediately preceding the wave reporting survival status (Table S3 in the **Online Supplementary Document**). We defined the patterns as: ‘Sustained non-engagement’ (*i.e.* No hobby engagement in either of the two exposure waves preceding the survival status assessment wave), ‘Initiation of engagement’ (*i.e.* No hobby engagement in the first exposure wave, but hobby engagement in the second exposure wave), ‘Cessation of engagement’ (*i.e.* Hobby engagement in the first exposure wave, but no hobby engagement in the second exposure wave), and ‘Sustained engagement’ (*i.e.* Hobby engagement in both exposure waves).

### Assessment of mortality

Mortality data (*e.g.* death date and cause of death) were retrieved from national mortality registers or end-of-life interviews with a proxy respondent, usually a family member or a next-of-kin [19–21,23]. Survival time was measured in years from the date of the interview to the time of death, loss to follow-up, or the end of the study period, whichever came first (Table S4 in the **Online Supplementary Document**) [24,25].

### Statistical analyses

Descriptive statistics were calculated for the baseline characteristics of participants in each country, with continuous variables presented as median and interquartile range (IQR) and categorical variables as numbers and percentages.

Primary analyses were conducted separately for each country before pooling results. We calculated all-cause mortality rates (per 1000 person-years) by dividing the total number of deaths by the total number of person-years of observation. Cox proportional hazards models were used to estimate the association of hobby engagement with all-cause mortality risk, adjusting for baseline covariates, with hazard ratios (HRs) and 95% confidence intervals (CIs) calculated and reported. The *P*-values for all HRs were based on Wald χ^2^ tests (Methods S2 and Table S5 in the **Online Supplementary Document**).

To account for potential selection bias due to loss to follow-up and mitigate time-varying confounding, we referred to similar research [22] to implement a marginal structural model (MSM) to analyse the impact of four change patterns of hobby engagement on all-cause mortality risk. This approach is ideal for estimating the effects of time-varying exposures and considering time-varying confounders and selective attrition in longitudinal data [26] (Methods S3 and Table S6 in the **Online Supplementary Document**).

Referring to previous research [1], results were pooled into a cross-national meta-analysis using random-effects models to estimate overall effect sizes, pooled effect sizes, 95% CIs and *P* values derived from Z tests were reported.

To quantify the potential impact of these associations at the population level, we also calculated the population attributable fraction (PAF) with 95% CIs for mortality dependence on hobby engagement exposure in a covariate-adjusted model by referring to previous research [27]. In this analysis, the PAF represents the estimated proportion of deaths that could be prevented if all older adults in the population engaged in hobbies, assuming unmeasured confounding factors do not influence the observed association. We also calculated subgroup and seven sensitivity analyses (Methods S4 in the **Online Supplementary Document**).

We did all analyses using STATA, version 15.0 (Stata Corp LLC, College Station, Texas, USA) except for MSM-Cox analysis done in *R*, version 4.0.4 (R Core Team, Vienna, Austria). We considered two-sided *P*-values <0.05 statistically significant.

## RESULTS

### Participant characteristics

Our analysis included 79 464 participants aged ≥50 across 19 countries, including 13 235 from the USA, 8143 from England, 11 322 from China, and 46 764 from 16 European countries. The hobby engagement level varied substantially across regions. European countries generally showed high engagement levels (84.2%), with particularly high levels in the Netherlands (97.3%), Sweden (97.1%), and Denmark (96.9%). The USA reported moderate engagement (55.3%), while China had the lowest participation levels (23.3%) (**Table 1**).

**Table 1:** Basic demographics by country*

	USA	England	China	Europe	Austria	Belgium	Czech Republic	Denmark	Estonia	France	Germany	Hungary	Italy	Netherlands	Poland	Portugal	Slovenia	Spain	Sweden	Switzerland
**Participants, n**	13 235	8143	11 322	46 764	4161	4156	4482	1999	6278	4309	1035	1896	2915	2261	1458	1591	2274	3249	1646	3054
**Hobby engagement**																				
Yes	7318 (55.3)	6445 (79.1)	2637 (23.3)	39 354 (84.2)	3850 (92.5)	3824 (92.0)	4042 (90.2)	1938 (96.9)	5677 (90.4)	3683 (85.5)	956 (92.4)	1517 (80.0)	1730 (59.3)	2200 (97.3)	902 (61.9)	940 (59.1)	1671 (73.5)	1914 (58.9)	1598 (97.1)	2912 (95.4)
No	5917 (44.7)	1698 (20.9)	8685 (76.7)	7410 (15.8)	311 (7.5)	332 (8.0)	440 (9.8)	61 (3.1)	601 (9.6)	626 (14.5)	79 (7.6)	379 (20.0)	1185 (40.7)	61 (2.7)	556 (38.1)	651 (40.9)	603 (26.5)	1335 (41.1)	48 (2.9)	142 (4.6)
**Age, MD (IQR)**	68 (60–76)	63 (56–72)	60 (56–67)	65 (58–73)	65 (58–72)	63 (56–73)	65 (59–71)	63 (56–71)	66 (58–74)	64 (57–74)	66 (60–72)	63 (58–71)	66 (60–73)	64 (58–71)	65 (60–74)	64 (57–71)	64 (57–73)	67 (59–76)	68 (63–75)	64 (57–72)
**Gender**																				
Men	5450 (41.2)	3815 (46.9)	5548 (49.0)	20 474 (43.8)	1779 (42.8)	1871 (45.0)	1884 (42.0)	931 (46.6)	2527 (40.3)	1878 (43.6)	481 (46.5)	802 (42.3)	1334 (45.8)	999 (44.2)	634 (43.5)	701 (44.1)	980 (43.1)	1508 (46.4)	749 (45.5)	1416 (46.4)
Women	7785 (58.8)	4328 (53.1)	5774 (51.0)	26 290 (56.2)	2382 (57.2)	2285 (55.0)	2598 (58.0)	1068 (53.4)	3751 (59.7)	2431 (56.4)	554 (53.5)	1094 (57.7)	1581 (54.2)	1262 (55.8)	824 (56.5)	890 (55.9)	1294 (56.9)	1741 (53.6)	897 (54.5)	1638 (53.6)
**Education level**																				
Less than lower secondary education	2276 (17.2)	3498 (43.0)	10 182 (89.9)	19 679 (42.1)	1018 (24.5)	1735 (41.7)	2138 (47.7)	348 (17.4)	1960 (31.2)	1913 (44.4)	117 (11.3)	612 (32.3)	2087 (71.6)	1045 (46.2)	623 (42.7)	1289 (81.0)	793 (34.9)	2666 (82.1)	717 (43.6)	618 (20.2)
Upper secondary and vocational training	7910 (59.8)	3571 (43.9)	961 (8.5)	17 826 (38.1)	2054 (49.4)	1120 (26.9)	1801 (40.2)	799 (40.0)	2990 (47.6)	1510 (35.0)	568 (54.9)	996 (52.5)	651 (22.3)	612 (27.1)	727 (49.9)	155 (9.7)	1112 (48.9)	310 (9.5)	470 (28.6)	1951 (63.9)
Tertiary education	3049 (23.0)	1074 (13.2)	179 (1.6)	9259 (19.8)	1089 (26.2)	1301 (31.3)	543 (12.1)	852 (42.6)	1328 (21.2)	886 (20.6)	350 (33.8)	288 (15.2)	177 (6.1)	604 (26.7)	108 (7.4)	147 (9.2)	369 (16.2)	273 (8.4)	459 (27.9)	485 (15.9)
**Marital status**																				
Married/partnered	8521 (64.4)	5821 (71.5)	9669 (85.4)	34 602 (74.0)	2798 (67.2)	2975 (71.6)	3180 (71.0)	1542 (77.1)	4318 (68.8)	2968 (68.9)	853 (82.4)	1339 (70.6)	2418 (83.0)	1849 (81.8)	1131 (77.6)	1291 (81.1)	1693 (74.5)	2621 (80.7)	1270 (77.2)	2356 (77.1)
Single	4714 (35.6)	2322 (28.5)	1653 (14.6)	12 162 (26.0)	1363 (32.8)	1181 (28.4)	1302 (29.0)	457 (22.9)	1960 (31.2)	1341 (31.1)	182 (17.6)	557 (29.4)	497 (17.0)	412 (18.2)	327 (22.4)	300 (18.9)	581 (25.5)	628 (19.3)	376 (22.8)	698 (22.9)
**Smoke**																				
Current smoker	1770 (13.4)	1391 (17.1)	3558 (31.4)	8578 (18.3)	817 (19.6)	775 (18.6)	1005 (22.4)	453 (22.7)	1243 (19.8)	665 (15.4)	180 (17.4)	410 (21.6)	479 (16.4)	403 (17.8)	339 (23.3)	150 (9.4)	319 (14.0)	491 (15.1)	223 (13.5)	626 (20.5)
Non-current smoker	11 465 (86.6)	6752 (82.9)	7764 (68.6)	38 186 (81.7)	3344 (80.4)	3381 (81.4)	3477 (77.6)	1546 (77.3)	5035 (80.2)	3644 (84.6)	855 (82.6)	1486 (78.4)	2436 (83.6)	1858 (82.2)	1119 (76.7)	1441 (90.6)	1955 (86.0)	2758 (84.9)	1423 (86.5)	2428 (79.5)
**Drink**																				
Yes	7161 (54.1)	7289 (89.5)	4704 (41.5)	21 065 (45.0)	2083 (50.1)	2575 (62.0)	1612 (36.0)	1408 (70.4)	1144 (18.2)	2487 (57.7)	533 (51.5)	518 (27.3)	1373 (47.1)	1451 (64.2)	189 (13.0)	762 (47.9)	828 (36.4)	1220 (37.6)	889 (54.0)	1993 (65.3)
No	6074 (45.9)	854 (10.5)	6618 (58.5)	25 699 (55.0)	2078 (49.9)	1581 (38.0)	2870 (64.0)	591 (29.6)	5134 (81.8)	1822 (42.3)	502 (48.5)	1378 (72.7)	1542 (52.9)	810 (35.8)	1269 (87.0)	829 (52.1)	1446 (63.6)	2029 (62.4)	757 (46.0)	1061 (34.7)
**Employment**																				
Employed	4953 (37.4)	2847 (35.0)	6620 (58.5)	12 176 (26.0)	909 (21.8)	1138 (27.4)	976 (21.8)	859 (43.0)	2012 (32.0)	1144 (26.5)	278 (26.9)	359 (18.9)	528 (18.1)	674 (29.8)	184 (12.6)	360 (22.6)	399 (17.5)	619 (19.1)	467 (28.4)	1270 (41.6)
Unemployed	8282 (62.6)	5296 (65.0)	4702 (41.5%)	34 588 (74.0)	3252 (78.2)	3018 (72.6)	3506 (78.2)	1140 (57.0)	4266 (68.0)	3165 (73.5)	757 (73.1)	1537 (81.1)	2387 (81.9)	1587 (70.2)	1274 (87.4)	1231 (77.4)	1875 (82.5)	2630 (80.9)	1179 (71.6)	2784 (58.4)
**Depressive symptoms**																				
Yes	2665 (20.1)	1896 (23.3)	4401 (38.9)	13 794 (29.5)	839 (20.2)	1249 (30.1)	1157 (25.8)	316 (15.8)	2578 (41.1)	1466 (34.0)	251 (24.3)	740 (39.0)	929 (31.9)	403 (17.8)	606 (41.6)	648 (40.7)	596 (26.2)	1138 (35.0)	311 (18.9)	567 (18.6)
No	10 570 (79.9)	6247 (76.7)	6921 (61.1)	32 970 (70.5)	3322 (79.8)	2907 (69.9)	3325 (74.2)	1683 (84.2)	3700 (58.9)	2843 (66.0)	784 (75.7)	1156 (61.0)	1986 (68.1)	1858 (82.2)	852 (58.4)	943 (59.3)	1678 (73.8)	2111 (65.0)	1335 (81.1)	2487 (81.4)
**ADL impaired**																				
Yes	2239 (16.9)	1567 (19.2)	2127 (18.8)	5526 (11.8)	407 (9.8)	613 (14.7)	430 (9.6)	133 (6.7)	1070 (17.0)	503 (11.7)	121 (11.7)	262 (13.8)	292 (10.0)	137 (6.1%)	252 (17.3)	279 (17.5)	233 (10.2)	447 (13.8)	171 (10.4)	176 (5.8)
No	10 996 (83.1)	6576 (80.8)	9195 (81.2)	41 238 (88.2)	3754 (90.2)	3543 (85.3)	4052 (90.4)	1866 (93.3)	5208 (83.0)	3806 (88.3)	914 (88.3)	1634 (86.2)	2623 (90.0)	2124 (93.9)	1206 (82.7)	1312 (82.5)	2041 (89.8)	2802 (86.2)	1475 (89.6)	2878 (94.2)
**Chronic illness**																				
≥1	9729 (73.5)	4197 (51.5)	5116 (45.)	27 872 (59.6)	2286 (54.9)	2380 (57.3)	3018 (67.3)	1100 (55.0)	4101 (65.3)	2326 (54.0)	702 (67.8)	1312 (69.2)	1800 (61.7)	1267 (56.0)	976 (66.9)	937 (58.9)	1289 (56.7)	1996 (61.4)	1009 (61.3)	2373 (45.0)
None	3506 (26.5)	3946 (48.5)	6206 (54.8)	18 892 (40.4)	1875 (45.1)	1776 (42.7)	1464 (32.7)	899 (45.0)	2177 (34.7)	1983 (46.0)	333 (32.2)	584 (30.8)	1115 (38.3)	994 (44.0)	482 (33.1)	654 (41.1)	985 (43.3)	1253 (38.6)	637 (38.7)	1681 (55.0)

ADL – activities of daily living, IQR – interquartile range, MD – median

*Presented as n (%) unless specified otherwise.

Across all cohorts, participants who engaged in hobbies were significantly younger, better educated, more likely to be married/partnered, current non-smokers, and had better health conditions (Tables S7–10 in the **Online Supplementary Document**). Furthermore, the baseline characteristics of the included and excluded participants differed slightly across cohorts (Tables S11–14 in the **Online Supplementary Document**).

### Association of hobby engagement with all-cause mortality

**Figure 1:**
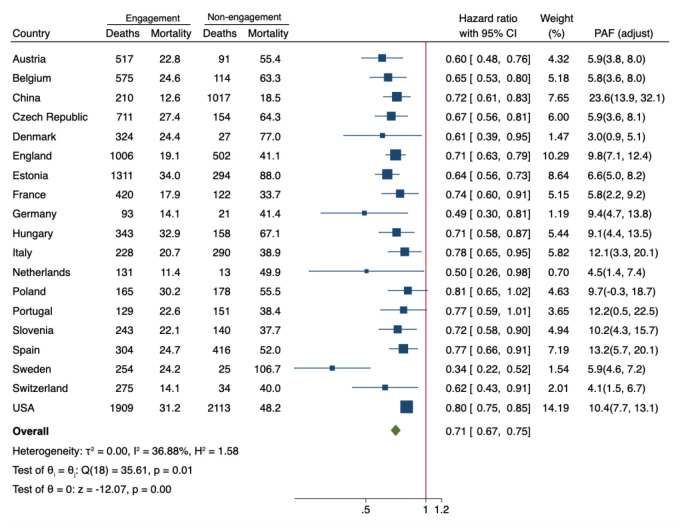
The association between hobby engagement and all-cause mortality risk across countries. Hazard ratio and PAF were adjusted for age, gender, education level, marital status, smoking, drinking, labour force status, depressive symptoms, activities of daily living impaired, presence of chronic illness. CI – confidence interval, PAF – population attributable fraction.

During a total of 536 539 person-years of follow-up (range = 0.04–13.28), 15 008 (18.9%) deaths were identified, including 9148 deaths among those who engaged in hobbies and 5860 among those who did not (**Figure 1**). The crude mortality rate was 23.96 (95% CI = 23.47, 24.46) per 1000 person-years in the hobby engagement group (ranging from 11.38 in the Netherlands to 33.98 in Estonia) and 37.87 (95% CI = 36.91, 38.85) per 1000 person-years in the non-engagement group (ranging from 18.47 in China to 106.69 in Sweden).

Overall, hobby engagement was associated with a 29% lower risk of all-cause mortality after accounting for a range of socio-demographic background, health behavioural, and health status confounders (pooled HR = 0.71; 95% CI = 0.67, 0.75) (**Figure 1**). The analysis revealed moderate heterogeneity across countries (*I*^2^ = 36.88%).

This protective association was consistently observed in most regions. The magnitude of this association varied across countries, with Sweden showing the strongest protective effect (HR = 0.34; 95% CI = 0.22, 0.52) and Italy demonstrating a more modest but still significant association (HR = 0.78; 95% CI = 0.65, 0.95). Among the 19 countries studied, only Poland (*P* = 0.07) and Portugal (*P* = 0.055) did not demonstrate statistically significant associations between hobby engagement and mortality risk.

PAFs analysis (**Figure 1**) revealed that, assuming residual free confounding, universal hobby engagement could potentially prevent 10.4% (weighted mean (x̄)) of all-cause mortality across the studied countries. The preventable fraction varied substantially between nations, ranging from 3.03% in Denmark to 23.56% in China. Notably, five countries demonstrated PAFs exceeding 10%: the USA (10.44%), Italy (12.12%), Slovenia (10.16%), Spain (13.20%), and China (23.56%), while England showed a PAF of 9.82%.

### Association of hobby engagement with life expectancy

Hobby engagement was associated with an estimated gain of 0.27 years (95% CI = 0.21, 0.34) in life expectancy during the five-year follow-up period across all studied countries, with substantial regional variations (**Table 2**). The greatest gains were observed in Sweden (x̄ = 1.02; 95% CI = 0.52, 1.53), Denmark, and Estonia (both exceeding 0.5 years), while more modest gains were seen in Europe (x̄ = 0.29; 95% CI = 0.26, 0.32), England (x̄ = 0.22; 95% CI = 0.18, 0.27), the USA (x̄ = 0.19; 95% CI = 0.16, 0.22), and China (x̄ = 0.06; 95% CI = 0.04, 0.09).

**Table 2:** Mean years of life saved among participants who engaged in the hobby up to 5 years by countries

	Survival years, x̄ (95% CI)			
	**Hobby engagement**	**Hobby non-engagement**	**Years of life saved, x̄ (95% CI)**	***P*-value**
**USA**	4.79 (4.77, 4.81)	4.60 (4.57, 4.63)	0.19 (0.16, 0.22)	<0.001
**England**	4.83 (4.82, 4.85)	4.61 (4.56, 4.65)	0.22 (0.18, 0.27)	<0.001
**China**	4.89 (4.87, 4.91)	4.83 (4.81, 4.84)	0.06 (0.04, 0.09)	<0.001
**Europe**	4.80 (4.80, 4.81)	4.52 (4.49, 4.54)	0.29 (0.26, 0.32)	<0.001
Austria	4.82 (4.79, 4.84)	4.45 (4.30, 4.59)	0.37 (0.23, 0.52)	<0.001
Belgium	4.83 (4.81, 4.85)	4.55 (4.43, 4.67)	0.28 (0.16, 0.40)	<0.001
Czech Republic	4.76 (4.73, 4.78)	4.33 (4.21, 4.46)	0.42 (0.29, 0.55)	<0.001
Denmark	4.78 (4.75, 4.82)	4.27 (3.95, 4.59)	0.51 (0.19, 0.83)	0.002
Estonia	4.73 (4.71, 4.76)	4.16 (4.04, 4.28)	0.58 (0.46, 0.70)	<0.001
France	4.84 (4.82, 4.86)	4.68 (4.60, 4.75)	0.16 (0.08, 0.24)	<0.001
Germany	4.87 (4.83, 4.91)	4.73 (4.52, 4.93)	0.14 (−0.07, 0.35)	0.182
Hungary	4.78 (4.74, 4.81)	4.50 (4.39, 4.62)	0.27 (0.15, 0.39)	<0.001
Italy	4.85 (4.82, 4.88)	4.61 (4.55, 4.67)	0.24 (0.17, 0.31)	<0.001
Netherlands	4.89 (4.87, 4.92)	4.49 (4.16, 4.82)	0.40 (0.07, 0.73)	0.017
Poland	4.72 (4.66, 4.78)	4.48 (4.38, 4.58)	0.24 (0.12, 0.35)	<0.001
Portugal	4.83 (4.79, 4.88)	4.75 (4.69, 4.81)	0.08 (0.004, 0.16)	0.04
Slovenia	4.84 (4.81, 4.87)	4.65 (4.57, 4.73)	0.19 (0.10, 0.28)	<0.001
Spain	4.78 (4.74, 4.81)	4.46 (4.39, 4.53)	0.32 (0.24, 0.39)	<0.001
Sweden	4.80 (4.76, 4.83)	3.77 (3.27, 4.27)	1.02 (0.52, 1.53)	<0.001
Switzerland	4.86 (4.84, 4.89)	4.52 (4.32, 4.72)	0.35 (0.14, 0.55)	0.001
**Pooled**	4.82 (4.79, 4.84)	4.53 (4.45, 4.62)	0.27 (0.21, 0.34)	<0.001

CI – confidence interval, x̄ – mean

### Association of hobby engagement with cause-specific mortality

Competing-risks regression analyses revealed distinct patterns of association between hobby engagement and cause-specific mortality across different cohorts (**Table 3**).

**Table 3:** The association of hobby engagement with cause-specific mortality risk across country/regional cohorts

	Hobby engagement		Hobby non-engagement			
	**Deaths**	**Crude mortality**†	**Deaths**	**Crude mortality**†	SHR (95% CI)*	***P-*value**
**USA (HRS)**						
Cancer	234	3.83	187	4.27	1.10 (0.90, 1.35)	0.33
Musculoskeletal disease	15	0.25	25	0.57	0.70 (0.36, 1.35)	0.29
Cardiovascular disease	276	4.51	267	6.10	1.04 (0.87, 1.24)	0.67
Respiratory disease	109	1.78	110	2.51	1.00 (0.76, 1.32)	0.99
Endocrine, metabolic, and nutritional conditions	9	0.15	28	0.64	0.31 (0.14, 0.69)	<0.001
Digestive system disease	74	1.21	57	1.30	1.28 (0.88, 1.86)	0.2
Neurological disease	22	0.36	43	0.98	0.51 (0.30, 0.87)	0.01
Mental and behavioural disorders	5	0.08	8	0.18	0.46 (0.16, 1.29)	0.14
**England (ELSA)**						
Cancer	186	3.54	53	4.34	1.13 (0.82, 1.55)	0.45
Cardiovascular disease	118	2.24	83	6.80	0.56 (0.42, 0.76)	<0.001
Respiratory disease	66	1.26	23	1.88	1.31 (0.79, 2.19)	0.3
**Europe (SHARE)**						
Cancer	1550	6.16	416	9.53	0.97 (0.85, 1.09)	0.57
A heart attack	588	2.34	261	5.98	0.77 (0.65, 0.91)	<0.001
A stroke	505	2.01	241	5.52	0.62 (0.51, 0.74)	<0.001
Other cardiovascular related illness	796	3.17	321	7.35	0.82 (0.71, 0.95)	0.01
Respiratory disease	248	0.99	154	3.53	0.68 (0.53, 0.87)	<0.001
Digestive system disease	160	0.64	55	1.26	0.97 (0.68, 1.39)	0.87
Severe infectious disease	254	1.01	110	2.52	0.76 (0.57, 1.01)	0.06
Accident	145	0.58	44	1.01	0.84 (0.57, 1.24)	0.38

CI – confidence interval, ELSA – English Longitudinal Study of Ageing, HRS – Health and Retirement Study, SHARE – Survey of Health, Ageing and Retirement in Europe, SHR – subhazard ratio

*Model was adjusted for age, gender, education level, marital status, smoke, drink, labour force status, depressive symptoms, activities of daily living impaired, presence of chronic illness. Country was further adjusted in SHARE.

†Per 1000 person-years.

In the US cohort, hobby engagement was significantly associated with reduced mortality risk from endocrine, metabolic and nutritional conditions (SHR = 0.31; 95% CI = 0.14, 0.69) and neurological disease (SHR = 0.51; 95% CI = 0.30, 0.87), while showing no significant associations with mortality risk from cancer, musculoskeletal disease, cardiovascular disease, respiratory disease, digestive system disease, or mental and behavioural disorders (*P* > 0.05).

The England cohort demonstrated a significant protective association between hobby engagement and cardiovascular disease mortality (SHR = 0.56; 95% CI = 0.42, 0.76), but no significant associations with cancer or respiratory disease mortality (*P* > 0.05).

In the European cohort, hobby engagement was significantly associated with reduced mortality risk from heart attack (SHR = 0.77; 95% CI = 0.65, 0.91), stroke (SHR = 0.62; 95% CI = 0.51, 0.74), other cardiovascular-related illnesses (SHR = 0.82; 95% CI = 0.71, 0.95), and respiratory disease (SHR = 0.68; 95% CI = 0.53, 0.83), while showing no significant associations with mortality from cancer, digestive system diseases, severe infectious diseases, or accidents (*P* > 0.05).

### Association between patterns of change in hobby engagement and mortality

**Figure 2:**
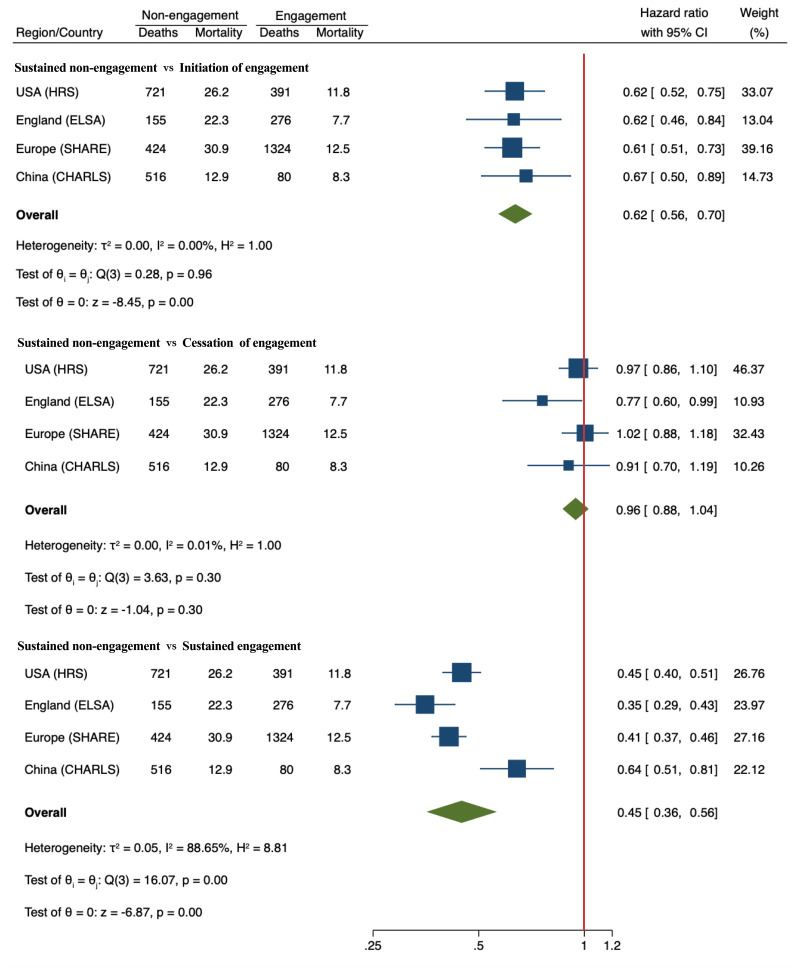
The association between four patterns of change in hobby engagement and all-cause mortality risk across country/regional cohorts. Hazard ratios were adjusted based on Marginal Structural Modelling Cox with both inverse probability of treatment weights and inverse probability of censoring weights applied. CHARLS – China Health and Retirement Longitudinal Study, CI – confidence interval, ELSA – English Longitudinal Study of Ageing, HRS – Health and Retirement Study, SHARE – Survey of Health, Ageing and Retirement in Europe.

Our multi-national data reveals substantial variations in hobby engagement patterns among adults aged ≥50 across countries (Table S15 in the **Online Supplementary Document**). Compared to sustained non-engagement, initiating hobby engagement was associated with a 38% reduction in all-cause mortality risk (pooled HR = 0.62; 95% CI = 0.56, 0.70; *I*^2^ = 0), while keeping hobby engagement showed an even stronger protective effect with a 55% reduction (pooled HR = 0.45; 95% CI = 0.36, 0.56; *I*^2^ = 88.65%) (**Figure 2**). This protective gradient was consistently observed across regions.

Notably, the mortality risk for respondents who ceased hobbies was comparable to those who sustained non-engagement in hobbies (pooled HR = 0.96; 95% CI = 0.88, 1.04; *P* = 0.3; *I*^2^ = 0.01%).

### Subgroup analysis

Interaction analyses showed that the negative association of hobby engagement on the all-cause mortality risk was more pronounced among older adults aged ≥65 than those aged 50–64 (pooled interaction HR = 0.79; 95% CI = 0.68, 0.91); and more pronounced among those who were married than those who were single (pooled interaction HR = 0.91; 95% CI = 0.85, 0.97) (Table S16 in the **Online Supplementary Document**).

The association between hobby engagement and mortality risk did not significantly differ across other demographic and health characteristics, including gender, education level, labour force status, depressive symptoms, and ADL impairment (all *P* > 0.05).

### Sensitivity analysis

Seven sensitivity analyses consistently supported our primary findings. Subgroup analyses by hobby measurement methods showed similar effect sizes between binary and listed measures (Table S17 in the **Online Supplementary Document**). The non-significant subgroup difference (*P* = 0.193) suggests that different assessment approaches across studies did not substantially influence the findings. The relatively large E-values across all cohorts suggest that substantial unmeasured confounding would be required to nullify our findings, supporting the robustness of the observed protective associations between hobby engagement and mortality risk (Table S18 in the **Online Supplementary Document**). Multiple imputation analyses for missing covariates, incorporation of survey weights, exclusion of participants with baseline chronic diseases, and additional adjustment for total household wealth yielded similar protective associations across all cohorts (Tables S19–22 in the **Online Supplementary Document**). Furthermore, the result of the meta-analysis that included only the three essentially aligned timeline cohorts (HRS, SHARE, and CHARLS) was consistent with the main analysis (Table S23 in the **Online Supplementary Document**).

## DISCUSSION

This first large-scale study of 19 countries provides robust evidence that hobby engagement is associated with a reduction in all-cause mortality risk of about three-tenths of one percent among adults aged ≥50, with PAFs suggesting that up to 23.56% of deaths might be preventable through universal hobby engagement in some regions. While previous research with 1853 Japanese older adults demonstrated increased mortality risk (HR = 1.66; 95% CI = 1.17, 2.35) among those who had purpose in life but lacked hobbies over three years [28], our study breaks new ground by demonstrating protective associations across dramatically different cultural contexts, from China to the USA, England, and diverse European nations. This multi-national validation establishes hobby engagement – beyond its potential interaction with life purpose – as a potentially universal modifiable factor for health promotion [2] – a critical insight previously unavailable from single-country studies. Alongside corresponding variations in potential life years saved (although not as considerable in some countries, such as China) and preventable mortality fractions, this underscores the substantial opportunity for targeted interventions while strengthening the evidence base for incorporating hobby promotion into global public health strategies for healthy ageing and longevity.

The potential mechanisms through which hobby engagement may be associated with lower mortality risk are multifaceted, encompassing psychological, biological, social, and behavioural processes [29]. Hobbies often involve imagination, novelty, creativity, and cognitive stimulation, which may contribute to enhanced cognitive reserve associated with increased longevity [7,8,10]. The sense of purpose and self-fulfilment often derived from hobbies may also contribute to improved immune function and healthier lifestyle choices [30]. From a physical health perspective, many hobbies, particularly exercise and outdoor activities, contribute to increased overall physical activity levels [31–33], potentially lowering the risk of cardiovascular diseases and other chronic conditions that significantly contribute to mortality in older populations [3]. The social interaction inherent in many hobbies enhances social capital (*e.g.* supportive networks) and reduces loneliness, factors consistently associated with a lower risk of mortality in older adults [34]. Furthermore, hobbies offer avenues for stress reduction, emotional well-being, and self-expression. These activities provide opportunities for relaxation and positive emotional experiences, which can alleviate chronic stress and depression [1]. By offering emotional, cognitive, and social coping resources, hobby engagement may support biological regulatory systems and promote behaviours associated with longevity [35]. The stronger associations between hobby engagement and lower mortality risk observed among adults aged ≥65 suggest hobbies become increasingly crucial with age.

The cause-specific mortality analyses revealed that hobby engagement was associated with lower rates of different causes of death across regions. The significant association between hobby engagement and reduced CVD mortality observed in England and Europe extends previous research, which had only examined specific hobby types such as sports activities or cultural pursuits concerning cardiovascular mortality in these regions [10,13,34,36]. Our findings demonstrate that the cardiovascular benefits may be attributed to overall hobby engagement rather than limited to physical activities or cultural pursuits [1,2], providing novel evidence for the broader potential of hobby engagement in cardiovascular health promotion [3,37]. However, there is an absence of a similar association between hobby engagement and lower rates of CVD mortality in the USA. This regional variation likely reflects complex interactions between hobby engagement and broader social contexts. Previous research indicates that the overall burden of cardiovascular risk factors differs across regions [38]. The USA has a higher prevalence of obesity and sedentary lifestyle than many European countries and the UK, which may counteract the protective associations of hobby engagement [39,40]. When hobby participation occurs against a backdrop of more substantial cardiovascular risk factors, the protective association between hobby engagement and cardiovascular disease mortality appears less significant. More comprehensive studies are needed to elucidate the complex relationship between hobby engagement and cardiovascular health in diverse national contexts.

In the USA, hobby participation was significantly associated with reduced mortality risk related to endocrine, metabolic, and nutritional conditions and neurological conditions. This finding builds upon earlier research that has demonstrated that cognitive and physical activities, often inherent in hobbies, may help manage metabolic health and preserve neurological function [41,42]. In Europe, hobby engagement was linked to a decreased mortality risk from respiratory diseases. The diverse protective associations observed across different regions suggest that while the overall benefit of hobby engagement is consistent, the pathways through which hobbies influence longevity may interact with local environmental, lifestyle, or health care factors [43]. Notably, the absence of significant associations with specific causes of death, such as cancer, across all cohorts indicates that the protective effects of hobby engagement may be specific to certain disease processes rather than universally applicable. These findings carry important public health implications, suggesting that promoting hobby engagement could be particularly effective in preventing particular causes of death, especially cardiovascular-related mortality.

In addition, our study advances beyond previous single-time point analyses by examining dynamic patterns of hobby engagement across multiple countries, revealing substantial variations in participation patterns. Sustained engagement showed the strongest association with lower mortality rates, with consistently engaged individuals having approximately half the mortality risk compared to non-engaged individuals. Initiating hobby engagement was also significantly associated with lower mortality risk. These benefits remained consistent across regions with dramatically different participation patterns, from China's predominantly non-engaged population (65.1% sustained non-engagement) to Denmark's near-universal participation (94.7% sustained engagement). Notably, our analysis revealed that individuals who ceased hobby engagement during the study period had mortality risks comparable to those who sustained non-engagement in hobbies, which may emphasise the importance of maintaining engagement over time. This approach considers hobby engagement as dynamic rather than static, accounting for potential reverse causality and changing health status over time [22,26], thus providing more robust estimates of the association between hobby patterns and mortality risk. Despite varying baseline participation rates, the consistency of these protective associations across different engagement patterns and cultural contexts strengthens the evidence for promoting both the initiation and maintenance of hobby engagement as a public health strategy [2,4].

Our findings, drawing from diverse national contexts ranging from Denmark's high sustained engagement (94.7%) to China's lower participation rates (16.0% sustained engagement), establish hobby promotion as a potentially universal and scalable approach to healthy longevity across global populations [2,4]. Dramatic variations in patterns of hobby engagement across countries highlight the need to tailor public health strategies to these different contexts, while the consistent protective associations across regions suggest the potential for global policy implementation. These insights call for a dual-focused approach [16,17] developing interventions to initiate hobby engagement where participation is low, while simultaneously creating supportive environments to maintain participation where engagement is already established. This could be achieved through integrating hobby promotion into existing health and social care systems via social prescribing [5,44], developing appropriate infrastructure for hobby activities, and fostering cross-cultural community networks that support sustained engagement [45]. Such comprehensive strategies should consider both regional participation patterns and national contexts to effectively promote hobby engagement as a global public health tool for healthy ageing [4].

However, several limitations of this study should be acknowledged. First, despite employing previously validated measurement approaches and maintaining relative consistency in questioning methods and binary indicator consolidation across countries [1], variations existed in hobby engagement assessment. Nevertheless, these measurement differences did not result in significant variations in the observed associations between hobbies and mortality risk. Second, the observational nature of this study limits causal inference. Third, due to ELSA data constraints, inconsistencies with the period of the other three cohorts may introduce sociodemographic differences that could influence the observed associations.

## CONCLUSIONS

This 19-country study demonstrates that hobby engagement was associated with a reduction in all-cause mortality risk of approximately three-tenths among older adults, with potential prevention of up to 23% of deaths and life expectancy gains of up to 1.02 years over five years. The associations between hobby engagement and lower mortality rates remained consistent across countries despite varying participation rates, with specific correlations observed between hobby engagement and lower rates of cardiovascular, metabolic, neurological, and respiratory mortality. Both initiating and sustaining hobby engagement showed associations with lower mortality risk, with sustained participation offering the most substantial survival benefits.

The substantial variations in hobby participation patterns across countries and corresponding differences in preventable mortality highlight significant opportunities for public health intervention. Policymakers should prioritise initiating and maintaining hobby engagement to promote healthy longevity globally, particularly in regions where current low engagement rates and higher PAFs suggest the greatest potential for survival benefits.

## Additional material


Online Supplementary Document
