# Supplementary material for: Hobby engagement and all-cause and cause-specific mortality risk among people aged 50 years and older in 19 countries
Source: J Glob Health. 2025 Jul 1;15:04181. doi: 10.7189/jogh.15.04181 (PMC12208283; doi:10.7189/jogh.15.04181)
Supplement: Online Supplementary Document [file jogh-15-04181-s001.pdf]

Supplement to: Guo Y, Yang F. Hobby engagement and all-cause and cause-specific mortality risk among people aged 50 years and older in 19 countries. J Glob Health. 2025;15:14181.

Supplementary Methods S1: Study design and participants

Supplementary Methods S2: Assessment of covariates

Supplementary Methods S3: The marginal structural model (MSM) and estimation of the weights.

Supplementary Methods S4: Statistical analyses

Table S1. Summary of study periods and final status of participant across four cohorts

Figure S1. Flow chart of participant inclusion and exclusion in the Health and Retirement Study (HRS).

Figure S2. Flow chart of participant inclusion and exclusion in the English Longitudinal Study of Ageing (ELSA).

Guo Y, Yang F. Hobby engagement and all-cause and cause-specific mortality risk among people aged 50 years and older in 19 countries. J Glob Health. 2025;15:14181.

Figure S3. Flow chart of participant inclusion and exclusion in the Survey of Health, Ageing and Retirement in Europe (SHARE).

Figure S4. Flow chart of participant inclusion and exclusion in the China Health and Retirement Longitudinal Study (CHARLS).

Table S2. Measurement of hobby engagement across four longitudinal cohorts

Table S3. The specific waves and years used to define the change patterns in hobby engagement

Table S4. Measurements of all-cause and cause-specific mortality across four longitudinal cohorts

Table S5. Harmonized definitions for covariates across four longitudinal cohorts

Table S6. Descriptive statistics of the stabilized combined total inverse probability weight trimmed at the 99% percentile stratified by cohort

Table S7. Comparison of Characteristics Between Hobby Engagement and Non-engagement in HRS

Table S8. Comparison of Characteristics Between Hobby Engagement and Non-engagement in ELSA

Table S9. Comparison of Characteristics Between Hobby Engagement and Non-engagement in SHARE

Table S10. Comparison of Characteristics Between Hobby Engagement and Non-engagement in CHARLS

Table S11: Comparison summary of baseline characteristics between the included participants and excluded participants in HRS

Table S12: Comparison summary of baseline characteristics between the included participants and excluded participants in ELSA

Table S13: Comparison summary of baseline characteristics between the included participants and excluded participants in SHARE

Table S14: Comparison summary of baseline characteristics between the included participants and excluded participants in CHARLS
Table S15. Frequency of patterns of change in hobby engagement across cohorts
Table S16. Subgroup and interaction analysis
Table S17. Subgroup analyses by hobbies measures in meta-analysis
Table S18. The E-value of association of hobby engagement and mortality.
Table S19. Cox proportional analyses with multiple imputations
Table S20. Cox proportional analyses with survey weight
Table S21. Cox proportional analyses excluding respondents with chronic illness (cancer, heart disease, stroke, diabetes, lung disease, or hypertension) at baseline
Table S22. Cox proportional analyses with further inclusion of total household wealth
Table S23. Meta-analysis of the association between hobby engagement and all-cause mortality risk across cohort studies (except the ELSA)

Supplementary Methods S1: Study design and participants

The Health and Retirement Study (HRS)

The HRS is a nationally representative, longitudinal survey of aging that includes more than 37,000 persons over the age of 50 and their spouses in 23,000 households in the United States. The study is sponsored by the National Institute on Aging and conducted by the Institute for Social Research at the University of Michigan. The HRS employs a sophisticated multi-stage area probability sampling design to ensure national representativeness [1]. The sampling process consists of four distinct stages[1,2], beginning with the selection of Primary Sampling Units (PSUs), which comprise U.S. Metropolitan Statistical Areas (MSAs) and non-MSA counties, using a Probability Proportionate to Size (PPS) selection method. The second stage involves the selection of area segments within these sampled PSUs, ensuring appropriate geographic stratification and clustering. The third stage commences with a complete enumeration of all housing units physically located within the selected Secondary Sampling Units (SSUs), followed by a systematic selection process from these comprehensive housing unit listings. The final stage involves the selection of household financial units within the sampled housing units. A key feature of the HRS sampling design is its deliberate oversampling of minority populations, specifically Black and Hispanic respondents, at an approximate ratio of 2:1. This oversampling strategy ensures adequate representation of minority groups for meaningful statistical analysis. The original HRS cohort was first interviewed in 1992 and has been followed up every two years since then, providing comprehensive data on demographics, health behaviors, physical and mental conditions, employment, income and assets. Since 2004, a rotating random 50% subgroup of respondents was invited to an enhanced interview and given a Leave Behind Psychosocial and Lifestyle Questionnaire (LBQ) to complete every four years, which incorporates detailed psychosocial and lifestyle factors[1]. The 2008 LBQ began to include questions on hobby participation, which had been defined and used in previous research [3]. The baseline correspondence rate for HRS was 78% [4]. The HRS monitors vital status by linking to the National Death Index, and a 98.8% validation of deaths with essentially zero false positives has previously been reported by HRS tracking studies [5]. The combination of robust study design, high response rates, comprehensive mortality tracking, and rigorous validation procedures makes HRS a high-quality data source for mortality research [5,6]. The latest ethical approval for HRS was obtained from the University of Michigan Institutional Review Board (IRB Protocol: HUM0061128).

For the current analysis, data from waves 9-15 of the HRS (2008-2020) were utilized, as wave 9 marked the introduction of consistent hobby engagement measurements. Referring to previous research [7], the study design allowed for the combination of two rotating half-samples, first interviewed about hobby engagement in 2008 (wave 9) and 2010 (wave 10) respectively, into a comprehensive dataset. The 2008 respondents were followed up in 2012 (wave 11), 2016 (wave 13), and 2020 (wave 15), while the 2010 respondents were followed up in 2014 (wave 12) and 2018 (wave 14). There were 8,133 and 10,734 respondents over the age of 50 who were given LBQ in Waves 9 and 10, totaling 18,867 potential participants. We excluded 4,545 participants due to missing baseline hobby engagement data, 614 participants who were lost to follow-up after their first survey, and an additional 473 participants due to missing data on key covariates. After

these exclusions, the final analytic sample consisted of 13,235 participants, of whom 4,022 died during the follow-up period. Figure S1 depicted participant inclusion and exclusion.

The English Longitudinal Study of Ageing (ELSA)

The ELSA is a large-scale, nationally representative study of individuals aged 50 and over and their partners living in private households in England. Initiated in 2002, the study's first round sample consisted of 11,391 respondents from respondents to the Health Survey for England (HSE) in 1998, 1999, and 2001, utilizing a multi-stage stratified random sampling procedure to ensure broad representation of the English population [8]. To summarize, the ELSA wave 1 sample was only selected from households that responded to HSE (Stage 2). Furthermore, households were only issued to field if they included at least one age-eligible individual (Stage 3) who, according to administrative records, remained alive (Stage 4) and gave permission to be re-contacted in the future (Stage 5). The study employs comprehensive data collection methods, including face-to-face interviews, self-completed questionnaires, and nurse visits every 4 years. With a household response rate of 70% and an individual response rate of 67% [4], ELSA demonstrates strong participant engagement. Its longitudinal design, with regular follow-ups every two years, collecting comprehensive data on demographics, health behaviors, physical and mental conditions, employment, income and assets, social and civic participation. The social and civic participation section contains questions on the respondents' hobby engagement, which has been defined and used in previous research [3]. Participants are followed up through linkage to the National Health Service central register, providing accurate mortality data [8].The latest mortality data are available as of February 2012. Both cross-sectional and longitudinal weights are available to adjust for non-response and calibrate to population distributions, further enhancing the study's reliability. This combination of rigorous sampling methodology, comprehensive data collection, and reliable mortality tracking through official registers positions ELSA as a high-quality data source for aging research, particularly for studies investigating mortality outcomes in older populations. Waves included in our study received ethical approvals from London Multi-Centre Research Ethics Committee (Wave 1: MREC/01/2/91, Wave 2: MREC/04/2/006, Wave 3: 05/MRE02/63), National Hospital for Neurology and Neurosurgery & Institute of Neurology Joint Research Ethics Committee (Wave 4: 07/H0716/48), Berkshire Research Ethics Committee (Wave 5: 09/H0505/124), NRES Committee South Central - Berkshire (Wave 6: 11/SC/0374).

For the current analysis, data from waves 1-6 of the ELSA (2002-2012) were utilized, as mortality data were only available for wave 6 at best. The initial sample included 11,522 participants aged 50 and above at baseline (wave 1). After excluding those with missing baseline hobby data (1,132), those lost to follow-up after the first survey (1,359), and missing covariate data (888), the final analytic sample consisted of 8,143 participants, of whom 1,508 died during the follow-up period. Figure S2 depicted participant inclusion and exclusion.

The Survey of Health, Ageing and Retirement in Europe (SHARE)

The SHARE is a comprehensive multinational longitudinal study focusing on individuals aged 50 and over and their partners, it strongly contributes to the understanding of the ageing process in Europe. Initiated in 2004 with 12 countries, SHARE has expanded to include 30 European nations by its eighth wave [9,10]. SHARE's robust design aims to draw inferences about the aging population across countries using probability-based sampling methods, adapting to each country's specific circumstances while maintaining international comparability. SHARE's sampling strategy is meticulously designed to ensure representativeness and efficiency [10]. Most countries employ a multi-stage stratified sampling approach, dividing the country into strata to represent different geographical areas and improve survey estimate efficiency. The study uses population registers with individual age information where available, implementing screening procedures when such data is unavailable. This attention to detail in sampling design underscores SHARE's commitment to producing high-quality, representative data. The initial wave in 2004 drew from over 50,000 addresses, achieving an average response rate of 62% at the household level [9]. Existing variation in performance over countries was for the most part consistent with previously known patterns from other international surveys. Conditional on household participation an interview could, on average, be obtained from more than 85% of eligible household members [9]. The SHARE collects extensive data on demographics, physical and mental health, behavioral risks, employment, assets, and activities through biennial core surveys. Previous research [3] have defined hobby engagement based on whether respondents engage in certain specific activities. In cases where a respondent has deceased, SHARE conducts end-of-life interviews with proxy respondents, who may be family members, household members, neighbors, or other close associates of the deceased. These interviews, conducted either in person or by phone, gather crucial information about the respondent's last year of life, including circumstances of death, time, and cause [10]. Previous research has extensively utilized SHARE's mortality data, and proxy responses from family members have been validated as an appropriate source of information for mortality studies [11,12]. The SHARE was approved by the Ethics Committee of the University of Mannheim and Ethics Council of the Max Planck Society (00006320).

For the current analysis, consistent with previous research [3], data from waves 4-8 of the SHARE (2010-2019) were utilized, as wave 4 started collecting questions about hobby engagement. The initial sample for this study consisted of 56,606 respondents aged 50 and above from Wave 4. We further excluded 1,019 participants due to missing baseline hobby engagement data, 8,176 participants who were lost to follow-up after their first survey, and an additional 647 participants due to missing data on key covariates. After these exclusions, the final analytic sample consisted of 46,764participants, of whom 8,251 died during the follow-up period. Figure S3 depicted participant inclusion and exclusion. The sample sizes for each country in the final analysis were as follows: Austria (n = 4,161), Belgium (n = 4,156), Czech Republic (n = 4,482), Denmark (n = 1,999), Estonia (n = 6,278), France (n = 4,309), Germany (n = 1,035), Hungary (n = 1,896), Italy (n = 2,915), Netherlands (n = 2,261), Poland (n = 1,458), Portugal (n = 1,591), Slovenia (n =

2,274), Spain (n = 3,249), Sweden (n = 1,646), and Switzerland (n = 3,054).

The China Health and Retirement Longitudinal Study (CHARLS)

The CHARLS is a nationally representative survey of adults aged 45 years and older and their spouses in mainland China. Initiated between June 2011 and March 2012, CHARLS encompasses 17,708 respondents from 150 counties across 28 provinces, with follow-up surveys conducted every two years. The study employs a rigorous multistage stratified probability-proportional-to-size sampling method, ensuring national representativeness [13]. CHARLS utilizes a four-step sampling process: First, 150 county-level units were randomly selected using probability-proportional-to-size, stratified by region, urban/rural status, and GDP per capita. Second, three primary sampling units (PSUs) were chosen from each county. Third, 24 households were randomly selected within each PSU. Finally, one individual aged 45 or older was randomly chosen from each household, with both the individual and their spouse interviewed. The survey collects comprehensive data on demographics, lifestyle, health conditions, employment, household economics, and social activities through in-depth, one-on-one interviews using a structured questionnaire. Previous research [3] have defined hobby engagement based on whether respondents engage in certain specific social activities. The survey's response rate was 80.5% [14], with participants followed up in 2013, 2015, and 2018. CHARLS maintains high data quality through its scientifically designed sampling frame, rigorous treatment of potential bias in non-response and sampling, and extensively tested questionnaire and field procedures. If a respondent’s death was reported during the follow-up, the CHARLS team attempted to identify a knowledgeable informant (typically a family member) and conducted exit interviews to obtain information about the death. As most Chinese older individuals lack extensive and reliable medical records, the CHARLS depends on proxy reports for the deceased, and it has been demonstrated that proxy replies from family members are acceptable for death studies [12,14]. The database provides weighted value variables to adjust for nonresponse and sampling-frame issues at each stage [13]. Before commencing the questionnaire, all participants gave their written informed consent and signed it. If the responder was illiterate, he or she would press the fingerprint after the interviewer narrated the consent document. The CHARLS was approved by the Biomedical Ethics Review Committee of Peking University (IRB00001052-14010).

For the current analysis, data from waves 1-4 of the CHARLS (2011-2018) were utilized. There were 13,641 participants aged 50 and over at baseline (Wave 1). We further excluded 1,091 participants due to missing baseline hobby engagement data, 599 participants who were lost to follow-up after their first survey, and an additional 629 participants due to missing data on key covariates. After these exclusions, the final analytic sample consisted of 11,322 participants, of whom 1,227 died during the follow-up period. Figure S4 depicted participant inclusion and exclusion.

Supplementary Methods S2: Assessment of covariates

Covariates that may confound the association between hobbies engagement and mortality risk were identified based on previous research [3]. In this study, time-constant covariates included respondents' self-reported gender (men or women) and education level (less than lower secondary education; upper secondary & vocational training; tertiary education). Time-varying covariates included age (continuous), marital status (married/partnered or single), smoke (current smoker or non-current smoker), drink (yes or no), labor force status (working or not working), depressive symptoms (yes or no), activities of daily living (ADLs) impaired (yes or no), and presence of chronic illness (one or more disease, or none) (Table S5 in the **Online Supplementary Document**).

Supplementary Methods S3: The marginal structural model (MSM) and estimation of the weights.

We employed a MSM to estimate the effect of changing patterns of hobby engagement on all-cause mortality risk by referring previous research [15,16], accounting for time-varying confounders and potential selection bias due to loss to follow-up. MSMs are a class of causal models that are ideal for estimating time-varying exposure effects in longitudinal data [17], and have been used for stronger causal explanations in observational studies [15,16,18]. In longitudinal observational studies with repeated measures of exposure and confounding factors, complex and dynamic relationships exist among variables. In these circumstances, bias may occur when a time-varying confounder, conditional on the past exposure history, is a predictor of subsequent exposure [17,19]. Meaning that time-varying confounders could influence subsequent values of a time-varying exposure or an outcome, and themselves could be influenced by prior values of exposure or other confounders [20]. The same holds true when a past, or time-varying, exposure history is an independent predictor of a subsequent time-varying confounder or an outcome. Furthermore, longitudinal studies often face the challenge of loss to follow-up, which can introduce selection bias [17,20]. Therefore, investigations of exposure-outcome relationships should properly account for these biases for accurate or less biased estimation.

In our longitudinal study, both change patterns of hobby engagement and most confounding factors were time-varying and repeated across multiple waves of follow-up. It is plausible that hobby engagement could be influenced by its own preceding values and those of baseline and time-varying confounders, while simultaneously affecting subsequent values of these confounders [19,20]. This creates complex and dynamic relationships among variables where traditional analytical approaches might introduce bias. Moreover, the sample of four cohorts in this study had varying degrees of dropout during the follow-up period. To account for all these potential biases, we calculated two types of weights: inverse probability of treatment weights (IPTW) to account for time-varying confounders, and inverse probability of censoring weights (IPCW) to adjust for potential selection bias due to dropout or loss to follow-up. The final MSM weights were derived by multiplying IPTW by IPCW. The stabilized inverse probability weight for individual i at

outcome wave k (survival outcomes were assessed from wave 3 to the last wave) was the product of the inverse probability of exposure to hobby engagement weight (IPTW) and the inverse probability of remaining uncensored weight (IPCW), incorporating adjustments for time-dependent confounding and censoring [15]. The estimates obtained from our MSM analysis can be interpreted as population average causal effects of change patterns of hobby engagement on all-cause mortality [17,20]. Research has shown that the results obtained from the MSM model in observational studies most closely approximate those from randomized controlled trials [16,17].

(1) Stabilized inverse probability of treatment weights:

$$SW_i^E(t) = \prod_{k=3} \frac{Pr\{A(k-1)|\bar{A}(k-2), L_0\}}{Pr\{A(k-1)|\bar{A}(k-2), \bar{L}_{k-2}\}}$$

Where *Pr{*}* in the above equation stands for the conditional probability function, $A(k-1)$ represents the time-varying independent variable (hobby engagement in this study) at time *k-1*, $\bar{A}(k-2)$ represents the hobby engagement history prior to time *k-1*, L_0 represents baseline covariates, $\bar{L}_{k-2}$ represents baseline and time-varying covariates up to time *k-2*.

Assessment of change in hobby engagement occurred only for living and participating respondents, so observations for outcome wave *k* required the participant to be alive and in the study at wave *k-1*, provided hobby engagement status at waves *k-1* and *k-2,* and had a survival outcome assessment at wave *k.* The equation's denominator is the probability that individual *i* had changed hobby engagement at wave *k-1*, given individual *i*'s hobby engagement and time-varying covariate values at time *k-2*, and baseline covariates. The numerator is the probability that individual *i* had changed hobby engagement at wave *k-1*, given individual *i*'s hobby engagement at time *k-2*, and baseline covariates.

Individual *i have* four change patterns of hobby engagement using above two exposure time points (from wave *k-2 to k-1):* (1) "*Sustained non-engagement*": No hobby engagement in either of the waves *k-1* and *k-2* preceding the survival outcome assessment at wave *k*; (2) "*Initiation of engagement*": No hobby engagement in the wave *k-2*, but hobby engagement in the wave *k-1*; (3) "*Cessation of engagement*": Hobby engagement in the wave *k-2*, but no hobby engagement in the wave *k-1*; (4) "*Sustained engagement*": Hobby engagement in both the waves *k-1* and *k-2*.

To calculate the IPTW, predicted probabilities from the numerator model were divided by the predicted probabilities from the denominator model. This provides a "stabilized" weight for each person *i*. The numerator part is added to "stabilize" the standard IPTWs as they tend to be highly variable. Hence, adding the numerator reduces variability in the weights and increases precision.

(2) Stabilized inverse probability of censoring weights:

$$SW_i^C(t) = \prod_{k=3} \frac{Pr\{C(k-1)|\bar{C}(k-2), L_0\}}{Pr\{C(k-1)|\bar{C}(k-2), \bar{L}_{k-2}\}}$$

The IPCW is proportional to the inverse of the probability of having withdrawn from the study through that follow-up wave, for each participant *i* and outcome wave *k*. The IPCW was estimated in the same manner as the IPTW, except that the pooled logistic models were fitted to output the probability of not experiencing censoring

A total IPW that incorporates both adjustment for time-dependent confounding and censoring was calculated by multiplying the IPTW x IPCW. Weights that deviate substantially from 1.0 indicate no positivity or misspecification of the model. Consistent with previous research [15], truncation to the 99th percentile eliminated bias from outlier weights. Table S9 shows the descriptive statistics of the total stabilized IPW in this study stratified by cohort and truncated to the 99th percentile.

Supplementary Methods S4: Statistical analyses

We compared the baseline characteristics of those with and without hobbies, as well as the baseline characteristics of included and excluded participants, using a Kruskal-Wallis test for continuous variables and a Pearson chi-square test for categorical variables. The assumption of proportional hazard was assessed using Schoenfeld residuals and was satisfied. For cause-specific mortality risk in relation to hobby engagement (except for CHARLS), we employed competing-risks regression based on Fine and Gray's proportional subhazards model with reference to previous research [21], reporting subhazard ratios (SHRs). This method accounts for competing events that prevent the outcome of interest. The meta-analysis was weighted to account for varying sample sizes across countries. Between-country heterogeneity was assessed using Cochran's Q and I^2 statistics, with $p<0.05$ for Q and $I^2>50\%$ indicating heterogeneity.

To contextualize our findings, we then refer to previous research [22] that calculated the years of life saved attributable to hobby engagement. We calculated the restricted mean survival time over a 5-year follow-up period using the area under the survival curves for both hobby-engaged and non-engaged participants. Years of life saved were estimated as the difference in life expectancy between the two groups. The 95% CI were derived using the upper and lower bounds of the expected survival curves, with p-values derived from Z tests.

Subgroup analyses were conducted by age (50-64 years, ≥65 years), gender, education level, marital status, labor force status, depressive symptoms, and ADL impaired. Interaction terms were tested using likelihood ratio tests to explore differences in the association between hobby engagement and all-cause mortality risk across different population subgroups.

Sensitivity analyses were performed to assess the robustness of our primary findings. These included: (1) Considering that the data may not be missing at random, the analyses were repeated

after multiple imputations of missing covariate values at baseline based on five replications and a chained equation approach. Linear and logistic regression methods were used for continuous and dichotomous or polytomous variables, respectively; (2) The analyses were repeated with the inclusion of sampling weights for each survey cohort, which considered differential selection probabilities and response rates; (3) Subgroup analyses by hobby measures explored whether differences in effect sizes might be attributable to varying hobby assessment methods across studies (binary measure or index created from a list of options); (4) To assess the potential impact of unmeasured confounding, we calculated E-values for our main findings. The E-value quantifies the minimum strength of association that an unmeasured confounder would need to have with both the exposure and the outcome to fully explain away the observed association, conditional on the measured covariates. (5) To further mitigate reverse causality, the analyze was repeated after exclusion of participants with chronic diseases (cancer, heart disease, stroke, diabetes, lung disease, or hypertension) at baseline. (6) Given that total household wealth data were missing for approximately 30% of participants in CHARLS, we conducted sensitivity analyses by repeating analyses with additional adjustment for total household wealth rather than including it in the main analyses. (7) To assess potential biases introduced by temporal differences, we conducted a separate meta-analysis excluding ELSA, using only the three cohorts with essentially aligned timelines.

Table S1. Summary of study periods and final status of participant across four cohorts

	HRS		ELSA	SHARE	CHARLS
Baseline wave	Wave 9 (Subsample 1): 2008/2009	Wave 10 (Subsample 2): 2010/2011	Wave 1: 2002/2003	Wave 4: 2011/2012	Wave 1: 2011/2012
Follow-up mortality data	Wave 11: 2012/2013	Wave 12: 2014/2015	Wave 2: 2004/2005	Wave 5: 2013	Wave 2: 2013/2014
	Wave 13: 2016/2017	Wave 14: 2018/2019	Wave 3: 2006/2007	Wave 6: 2015	Wave 3: 2015/2016
	Wave 15: 2020/2021		Wave 4: 2008/2009	Wave 7: 2017	Wave 4: 2018
			Wave 5: 2010/2011	Wave 8: 2019	
			Wave 6: 2012/2013		
Years of follow-up: mean (range)	7.93 (0.04-13.28)		7.96 (1-11)	6.31 (0.08-10.75)	6.33 (0.08-7.17)
Final status					
Alive, n (%)	7,558 (57.11%)		4,885 (59.99%)	19,190 (41.04%)	9,172 (81.01%)
Dead, n (%)	4,022 (30.39%)		1,508 (18.52%)	8,251 (17.64%)	1,227 (10.84%)
Drop out, n (%)	1,655 (12.50%)		1,750 (21.49%)	19,323 (41.32%)	923 (8.15%)

Note: HRS: Health and Retirement Study; ELSA: English Longitudinal Study of Ageing; SHARE: Survey of Health, Ageing and Retirement in Europe; CHARLS: China Health and Retirement Longitudinal Study.

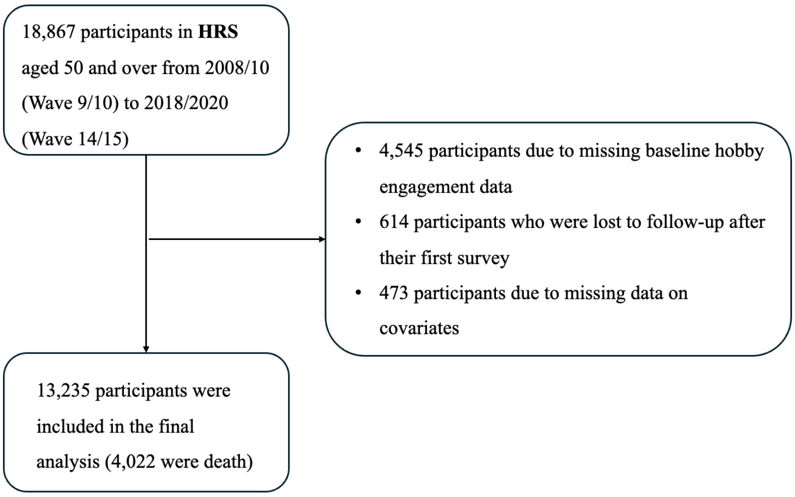

Figure S1. Flow chart of participant inclusion and exclusion in the Health and Retirement Study (HRS).

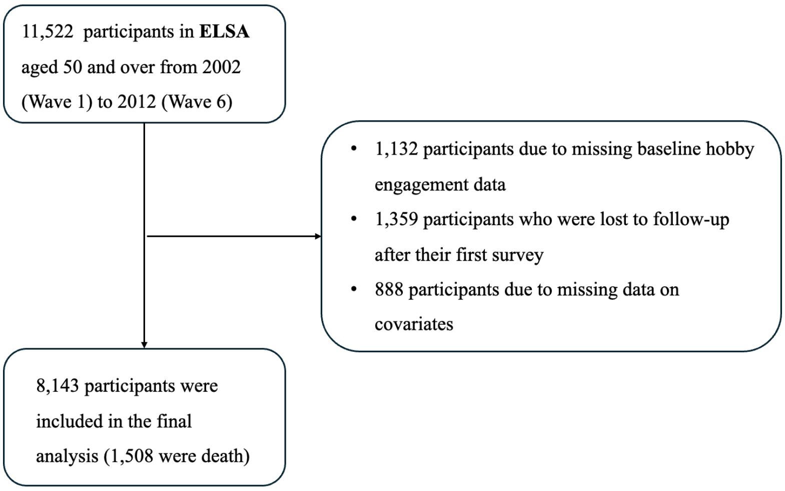

Figure S2. Flow chart of participant inclusion and exclusion in the English Longitudinal Study of Ageing (ELSA).

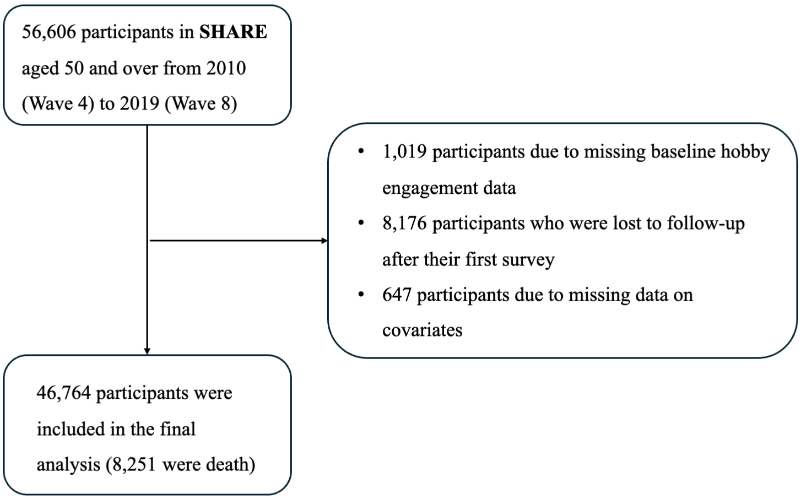

Figure S3. Flow chart of participant inclusion and exclusion in the Survey of Health, Ageing and Retirement in Europe (SHARE).

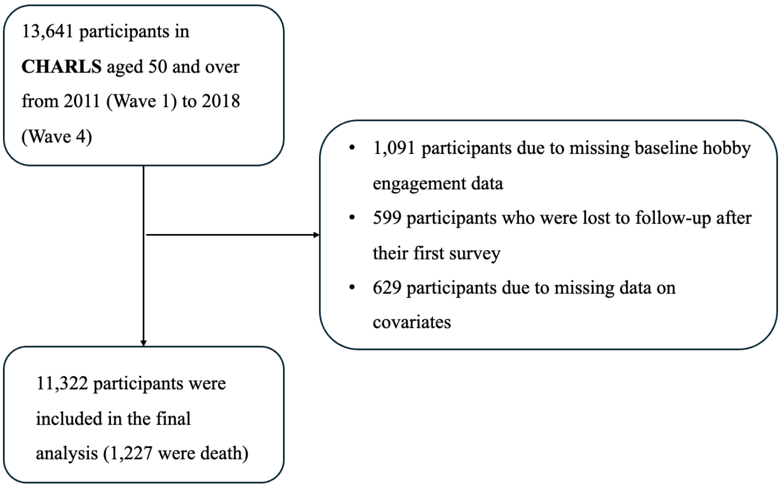

Figure S4. Flow chart of participant inclusion and exclusion in the China Health and Retirement Longitudinal Study (CHARLS).

Table S2. Measurement of hobby engagement across four longitudinal cohorts

Survey cohort	Questions	Cut-off value	Reference
HRS	I have a hobby or past time.	1=yes; 0=no	Mak, H.W., Noguchi, T., Bone, J.K. et al. Hobby engagement and mental wellbeing among people aged 65 years and older in 16 countries. Nat Med 29, 2233–2240 (2023). https://doi.org/10.1038/s41591-023-02506-1
ELSA	How often do you work on a hobby or project?	1=any frequency; 0=not in the last month/never	
SHARE	Which of the activities listed on this card – if any – have you done in the past twelve months?' 1. Done voluntary or charity work. 2. Attended an educational or training course. 3. Gone to a sport, social or other kind of club. 4. Taken part in a political or community-related organization. 5. Read books, magazines or newspapers. 6. Did word or number games such as crossword puzzles or Sudoku. 7. Played cards or games such as chess. 0. None of these	1=any of the activities; 0=none	
CHARLS	Which of the social activities listed on this card – if any – have you done in the past month?' 1. Played ma-jong, played chess, played cards or went to a community club. 2. Went to a sport, social or other kind of club. 3. Took part in a community-related organization. 4. Did voluntary or charity work. 5. Attended an educational or training course. 0. None of these	1=any of the activities; 0=none	

Note: HRS: Health and Retirement Study; ELSA: English Longitudinal Study of Ageing; SHARE: Survey of Health, Ageing and Retirement in Europe; CHARLS: China Health and Retirement Longitudinal Study.

Table S3. The specific waves and years used to define the change patterns in hobby engagement

**Survey cohort**	Assessment wave of change patterns in hobby engagement		Survival status assessment wave
	First exposure wave	Second exposure wave	
HRS	Wave 9 (Subsample 1): 2008/2009	Wave 11: 2012/2013	Wave 13: 2016/2017
	Wave 11 (Subsample 1): 2012/2013	Wave 13: 2016/2017	Wave 15: 2020/2021
	Wave 10 (Subsample 2): 2010/2011	Wave 12: 2014/2015	Wave 14: 2018/2019
ELSA	Wave 1: 2002/2003	Wave 2: 2004/2005	Wave 3: 2006/2007
	Wave 2: 2004/2005	Wave 3: 2006/2007	Wave 4: 2008/2009
	Wave 3: 2006/2007	Wave 4: 2008/2009	Wave 5: 2010/2011
	Wave 4: 2008/2009	Wave 5: 2010/2011	Wave 6: 2012/2013
SHARE	Wave 4: 2011/2012	Wave 5: 2013	Wave 6: 2015
	Wave 5: 2013	Wave 6: 2015	Wave 7: 2017
	Wave 6: 2015	Wave 7: 2017	Wave 8: 2019
CHARLS	Wave 1: 2011/2012	Wave 2: 2013/2014	Wave 3: 2015/2016
	Wave 2: 2013/2014	Wave 3: 2015/2016	Wave 4: 2018

Note: HRS: Health and Retirement Study; ELSA: English Longitudinal Study of Ageing; SHARE: Survey of Health, Ageing and Retirement in Europe; CHARLS: China Health and Retirement Longitudinal Study.

Table S4. Measurements of all-cause and cause-specific mortality across four longitudinal cohorts

**Survey cohort**	**Assessment of mortality**	**Available causes of death**
HRS	Mortality is determined through both household member reports and the National Death Index (NDI). A 98.8% validation of deaths with essentially zero false positives has previously been reported by HRS tracking studies [5]. The date of death was recorded.	• Cancer<br>• Musculoskeletal disease<br>• Cardiovascular disease<br>• Respiratory disease<br>• Endocrine, metabolic and nutritional conditions<br>• Digestive system disease<br>• Neurological disease<br>• Mental and behavioral disorders
ELSA	Data on all-cause mortality is retrieved from the National Health Service Central Data Registry [8]. Time of death is available by year.	• Cancer<br>• Cardiovascular disease<br>• Respiratory disease
SHARE	Mortality data are derived from end-of-life interviews with proxy respondents [9,10]. The interview is a special form of proxy interview conducted either in person or by phone. Proxy-respondents can be a family or household member, a neighbor or any other person of the closer social network of the deceased respondent. The end-of-life interview mainly contains information on respondent’s last year of life and the circumstance of death like time and cause of death. Previous research [11] has extensively utilized SHARE's mortality data, and proxy responses from family members have been validated as an appropriate source of information for mortality studies [23].	• Cancer<br>• A heart attack<br>• A stroke<br>• Other cardiovascular related illness<br>• Respiratory disease<br>• Disease of the digestive system<br>• Severe infectious disease<br>• Accident
CHARLS	Mortality data is collected through exit interviews with knowledgeable informants (usually a family member) when a respondent's death is reported [14]. As most Chinese older individuals lack	/

	extensive and reliable medical records, the CHARLS depends on proxy reports for the deceased, and it has been demonstrated that proxy replies from family members are acceptable for death studies[1]. The specific time of death is available in the 2013 wave; for other waves, the median time is used if exact dates are not available consistent with previous research [24,25] .	

Note: HRS: Health and Retirement Study; ELSA: English Longitudinal Study of Ageing; SHARE: Survey of Health, Ageing and Retirement in Europe; CHARLS: China Health and Retirement Longitudinal Study.

Table S5. Harmonized definitions for covariates across four longitudinal cohorts

Variables	Harmonized values	Definition in four surveys			
		HRS	ELSA	SHARE	CHARLS
Age [3]	Continuous	Age was recorded at the time of the interview with the inclusion of respondents aged 50 and over.			
Gender [3]	1= men; 0= women	The gender was reported as men or women.			
Education level [26]	1= Less than lower secondary education	Less than high school.		None, Primary education, or lower secondary education.	"No Formal Education (Illiterate)", "Did Not Finish Primary School but can Read", "Sishu (Private Tutoring)", "Elementary School" or "Middle School".
	2= Upper secondary and vocational training	GED, high-school graduate, or some college.	High-school graduate, or some college.	Upper secondary education, post-secondary non tertiary education.	"High School" or "Vocational School".
	3= Tertiary education	College and above.		First stage of tertiary education, or Second stage of tertiary education.	"Two/three-year College", "College Grad" or "Post-graduate degree".
Marital	1=	Married and living together with spouse; Partnered.			

status [27]	married/partnered			
	0= single	Separated, divorced, widowed, and never married.		
Smoke [26]	1= current smoker	Participants smoke at present.		
	0= non-current smoker	Participants had never smoked or had ever smoked but stopped at present.		
Drink [26]	1= Yes	Participants drink alcoholic at present or previously.	Participants drank alcohol or more weekly.	Participants drink alcoholic at present or previously.
	0= No	Participants never drunk alcoholic.	Participants drank alcohol less than weekly.	Participants never drunk alcoholic.
Labor force status [27]	1=Working	Respondents are currently working for pay or self-employment.		
	0=Not working	Respondents are currently retired or not working for other reasons.		
Depressive symptoms [27]	1=Yes	CES-D 8 scale, score range: 0-8; elevated symptoms (≥3).	Euro-D scale, score range: 0-12; elevated symptoms (≥4).	CES-D 10 scale, score range: 0-30; elevated symptoms (≥10).
	0=No	CES-D 8 <3	Euro-D <4	CES-D 10 <10
ADL impaired [27]	1=Yes	Participants answered with difficulty in at least one of the following daily activities: bathing, dressing, eating, getting in and out of bed, using the toilet, and walking across a room.		Daily activities including bathing, dressing, eating, getting in and out of bed, toileting, and controlling urination.

	0=No	Participants do not have any difficulties.	Participants do not have any difficulties.
Presence of chronic illness [6]	1=1 or more disease	Participants had been diagnosed with chronic conditions listed below by a doctor: cancer, heart problems, stroke, diabetes, lung disease, or hypertension.	
	0= None	Participants do not have any chronic diseases.	

Note: HRS: Health and Retirement Study; ELSA: English Longitudinal Study of Ageing; SHARE: Survey of Health, Ageing and Retirement in Europe; CHARLS: China Health and Retirement Longitudinal Study; GED: General Educational Development; CES-D: Center for Epidemiologic Studies Depression Scale; ADL: activities of daily living.

Table S6. Descriptive statistics of the stabilized combined total inverse probability weight trimmed at the 99% percentile stratified by cohort

Survey cohort	Mean	Standard deviation	Minimum	Maximum
HRS	1.00	0.03	0.63	1.10
ELSA	1.02	0.07	0.46	1.22
SHARE	0.98	0.09	0.45	1.29
CHARLS	1.00	0.07	0.60	1.28

Note: HRS: Health and Retirement Study; ELSA: English Longitudinal Study of Ageing; SHARE: Survey of Health, Ageing and Retirement in Europe; CHARLS: China Health and Retirement Longitudinal Study

Table S7. Comparison of Characteristics Between Hobby Engagement and Non-engagement in HRS

Characteristics	Hobby engagement	Hobby non-engagement	*P* value
Age (years)	67 (59-74)	69 (60-77)	< 0.001
Gender			
Men	3,222 (44.03%)	2,228 (37.65%)	< 0.001
Women	4,096 (55.97%)	3,689 (62.35%)	
Education level			
Less than lower secondary education	847 (11.57%)	1,429 (24.15%)	< 0.001
Upper secondary and vocational training	4,374 (59.77%)	3,536 (59.76%)	
Tertiary education	2,097 (28.66%)	952 (16.09%)	
Marital status			
Married/partnered	5,015 (68.53%)	3,506 (59.25%)	< 0.001
Single	2,303 (31.47%)	2,411 (40.75%)	
Smoke			
Current smoker	876 (11.97%)	894 (15.11%)	< 0.001
Non-current smoker	6,442 (88.03%)	5,023 (84.89%)	
Drink			
Yes	4,318 (59.01%)	2,843 (48.05%)	< 0.001
No	3,000 (40.99%)	3,074 (51.95%)	
Labor force status			
Working	2,986 (40.80%)	1,967 (33.24%)	< 0.001
Not working	4,332 (59.20%)	3,950 (66.76%)	
Depressive symptoms			
Yes	1,131 (15.46%)	1,534 (25.93%)	< 0.001
No	6,187 (84.54%)	4,383 (74.07%)	
ADL impaired			
Yes	901 (12.31%)	1,338 (22.61%)	< 0.001
No	6,417 (87.69%)	4,579 (77.39%)	
Presence of chronic illness			
1 or more disease	5,160 (70.51%)	4,569 (77.22%)	< 0.001
None	2,158 (29.49%)	1,348 (22.78%)	

Note: HRS: Health and Retirement Study; ADL: activities of daily living. Median (Q_{25}-Q_{75}) was used to describe continuous variables, and number (constituent ratio [%]) was used to describe categorical variables. The Kruskal-Wallis test for continuous variables and the Pearson chi-square test for categorical variables.

Table S8. Comparison of Characteristics Between Hobby Engagement and Non-engagement in ELSA

Characteristics	Hobby engagement	Hobby non-engagement	*P* value
Age (years)	62 (56-71)	66 (57-75)	< 0.001
Gender			
Men	3,053 (47.37%)	762 (44.88%)	0.07
Women	3,392 (52.63%)	936 (55.12%)	
Education level			
Less than lower secondary education	2,432 (37.73%)	1,066 (62.78%)	< 0.001
Upper secondary and vocational training	3,033 (47.06%)	538 (31.68%)	
Tertiary education	980 (15.21%)	94 (5.54%)	
Marital status			
Married/partnered	4,731 (73.41%)	1,090 (64.19%)	< 0.001
Single	1,714 (26.59%)	608 (35.81%)	
Smoke			
Current smoker	1,011 (15.69%)	380 (22.38%)	< 0.001
Non-current smoker	5,434 (84.31%)	1,318 (77.62%)	
Drink			
Yes	5,893 (91.44%)	1,396 (82.21%)	< 0.001
No	552 (8.56%)	302 (17.79%)	
Labor force status			
Working	2,385 (37.01%)	462 (27.21%)	< 0.001
Not working	4,060 (62.99%)	1,236 (72.79%)	
Depressive symptoms			
Yes	1,251 (19.41%)	645 (37.99%)	< 0.001
No	5,194 (80.59%)	1,053 (62.01%)	
ADL impaired			
Yes	1,066 (16.54%)	501 (29.51%)	< 0.001
No	5,379 (83.46%)	1,197 (70.49%)	
Presence of chronic illness			
1 or more disease	3,199 (49.64%)	998 (58.78%)	< 0.001
None	3,246 (50.36%)	700 (41.22%)	

Note: ELSA: English Longitudinal Study of Ageing; ADL: activities of daily living. Median (Q_{25}-Q_{75}) was used to describe continuous variables, and number (constituent ratio [%]) was used to describe categorical variables. The Kruskal-Wallis test for continuous variables and the Pearson chi-square test for categorical variables.

Table S9. Comparison of Characteristics Between Hobby Engagement and Non-engagement in SHARE

Characteristics	Hobby engagement	Hobby non-engagement	*P* value
Age (years)	64 (58-72)	68 (60-77)	< 0.001
Gender			
Men	17,213 (43.74%)	3,261 (44.01%)	0.7
Women	22,141 (56.26%)	4,149 (55.99%)	
Education level			
Less than lower secondary education	14,346 (36.45%)	5,333 (71.97%)	< 0.001
Upper secondary and vocational training	16,125 (40.97%)	1,701 (22.96%)	
Tertiary education	8,883 (22.57%)	376 (5.07%)	
Marital status			
Married/partnered	29,262 (74.36%)	5,340 (72.06%)	< 0.001
Single	10,092 (25.64%)	2,070 (27.94%)	
Smoke			
Current smoker	7,329 (18.62%)	1,249 (16.86%)	< 0.001
Non-current smoker	32,025 (81.38%)	6,161 (83.14%)	
Drink			
Yes	18,838 (47.87%)	2,227 (30.05%)	< 0.001
No	20,516 (52.13%)	5,183 (69.95%)	
Labor force status			
Working	11,087 (28.17%)	1,089 (14.70%)	< 0.001
Not working	28,267 (71.83%)	6,321 (85.30%)	
Depressive symptoms			
Yes	10,471 (26.61%)	3,323 (44.84%)	< 0.001
No	28,883 (73.39%)	4,087 (55.16%)	
ADL impaired			
Yes	3,906 (9.93%)	1,620 (21.86%)	< 0.001
No	35,448 (90.07%)	5,790 (78.14%)	
Presence of chronic illness			
1 or more disease	23,091 (58.68%)	4,781 (64.52%)	< 0.001
None	16,263 (41.32%)	2,629 (35.48%)	

Note: SHARE: Survey of Health, Ageing and Retirement in Europe; ADL: activities of daily living. Median (Q_{25}-Q_{75}) was used to describe continuous variables, and number (constituent ratio [%]) was used to describe categorical variables. The Kruskal-Wallis test for continuous variables and the Pearson chi-square test for categorical variables.

Table S10. Comparison of Characteristics Between Hobby Engagement and Non-engagement in CHARLS

Characteristics	Hobby engagement	Hobby non-engagement	*P* value
Age (years)	60 (55-66)	61 (56-67)	< 0.001
Gender			
Men	1,518 (57.57)	4,030 (46.40)	< 0.001
Women	1,119 (42.43)	4,655 (53.60)	
Education level			
Less than lower secondary education	2,128 (80.70)	8,054 (92.73)	< 0.001
Upper secondary and vocational training	413 (15.66)	548 (6.31)	
Tertiary education	96 (3.64)	83 (0.96)	
Marital status			
Married/partnered	2,332 (88.43)	7,337 (84.48)	< 0.001
Single	305 (11.57)	1,348 (15.52)	
Smoke			
Current smoker	976 (37.01)	2,582 (29.73)	< 0.001
Non-current smoker	1,661 (62.99)	6,103 (70.27)	
Drink			
Yes	1,317 (49.94)	3,387 (39.00)	< 0.001
No	1,320 (50.06)	5,298 (61.00)	
Labor force status			
Working	1,350 (51.19)	5,270 (60.68)	< 0.001
Not working	1,287 (48.81)	3,415 (39.32)	
Depressive symptoms			
Yes	705 (26.73%)	3,696 (42.56%)	< 0.001
No	1,932 (73.27%)	4,989 (57.44%)	
ADL impaired			
Yes	316 (11.98%)	1,811 (20.85%)	< 0.001
No	2,321 (88.02%)	6,874 (79.15%)	
Presence of chronic illness			
1 or more disease	1,282 (48.62%)	3,834 (44.15%)	< 0.001
None	1,355 (51.38%)	4,851 (55.85%)	

Note: CHARLS: China Health and Retirement Longitudinal Study; ADL: activities of daily living. Median (Q_{25}-Q_{75}) was used to describe continuous variables, and number (constituent ratio [%]) was used to describe categorical variables. The Kruskal-Wallis test for continuous variables and the Pearson chi-square test for categorical variables.

Table S11: Comparison summary of baseline characteristics between the included participants and excluded participants in HRS

**Characteristic**		**Included participants (n=13235)**	**Excluded participants (n=5632)**	*P*-value
Age		68 (60-76)	67 (57-77)	0.0001
Gender				
	Men	5450 (41.18%)	2452 (43.54%)	0.003
	Women	7785 (58.82%)	3180 (56.46%)	
Education level				
	Less than lower secondary education	2276 (17.2%)	1638 (29.1%)	<0.001
	Upper secondary and vocational training	7910 (59.77%)	2991 (53.14%)	
	Tertiary education	3049 (23.04%)	1000 (17.77%)	
Marital status				
	Married/partnered	8521 (64.38%)	3181 (56.5%)	<0.001
	Single	4714 (35.62%)	2449 (43.5%)	
Smoke				
	Current smoker	1770 (13.37%)	872 (15.85%)	<0.001
	Non-current smoker	11465 (86.63%)	4629 (84.15%)	
Drink				
	Yes	7161 (54.11%)	2693 (47.84%)	<0.001
	No	6074 (45.89%)	2936 (52.16%)	
Labor force status				
	Working	4953 (37.42%)	2347 (41.67%)	<0.001
	Not working	8282 (62.58%)	3285 (58.33%)	
Depressive symptoms				
	Yes	2665 (20.14%)	1222 (27.42%)	<0.001
	No	10570 (79.86%)	3235 (72.58%)	
ADL impaired				
	Yes	2239 (16.92%)	1517 (27.2%)	<0.001
	No	10996 (83.08%)	4060 (72.8%)	
Presence of chronic illness				
	1 or more disease	9729 (73.51%)	4168 (74.01%)	0.479
	None	3506 (26.49%)	1464 (25.99%)	

Note: HRS: Health and Retirement Study; ADL: activities of daily living. Median (Q_{25}-Q_{75}) was used to describe continuous variables, and number (constituent ratio [%]) was used to describe categorical variables. The Kruskal-Wallis test for continuous variables and the Pearson chi-square test for categorical variables.

Table S12: Comparison summary of baseline characteristics between the included participants and excluded participants in ELSA

**Characteristic**		**Included participants (n=8143)**	**Excluded participants (n=3379)**	*P*-value
Age		63 (56-72)	66 (57-75)	<0.001
Gender				
	Men	3815 (46.85%)	1415 (41.88%)	
	Women	4328 (53.15%)	1964 (58.12%)	
Education level				
	Less than lower secondary education	3498 (42.96%)	1402 (59.18%)	<0.001
	Upper secondary and vocational training	3571 (43.85%)	757 (31.95%)	
	Tertiary education	1074 (13.19%)	210 (8.86%)	
Marital status				
	Married/partnered	5821 (71.48%)	2162 (64.02%)	<0.001
	Single	2322 (28.52%)	1215 (35.98%)	
Smoke				
	Current smoker	1391 (17.08%)	628 (19.61%)	0.002
	Non-current smoker	6752 (82.92%)	2574 (80.39%)	
Drink				
	Yes	7289 (89.51%)	2704 (84.55%)	<0.001
	No	854 (10.49%)	494 (15.45%)	
Labor force status				
	Working	2847 (34.96%)	935 (28.15%)	<0.001
	Not working	5296 (65.04%)	2387 (71.85%)	
Depressive symptoms				
	Yes	1896 (23.28%)	849 (28.08%)	<0.001
	No	6247 (76.72%)	2174 (71.92%)	
ADL impaired				
	Yes	1567 (19.24%)	777 (24.27%)	<0.001
	No	6576 (80.76%)	2424 (75.73%)	
Presence of chronic illness				
	1 or more disease	4197 (51.54%)	1910 (56.64%)	<0.001
	None	3946 (48.46%)	1462 (43.36%)	

Note: ELSA: English Longitudinal Study of Ageing; ADL: activities of daily living. Median (Q_{25}-Q_{75}) was used to describe continuous variables, and number (constituent ratio [%]) was used to describe categorical variables. The Kruskal-Wallis test for continuous variables and the Pearson chi-square test for categorical variables.

Table S13: Comparison summary of baseline characteristics between the included participants and excluded participants in SHARE

**Characteristic**		**Included participants (n=46,764)**	**Excluded participants (n= 9,842)**	*P*-value
Age		65 (58-73)	64 (57-74)	0.0063
Gender				
	Men	20474 (43.78%)	4414 (44.85%)	0.053
	Women	26290 (56.22%)	5,428 (55.15%)	
Education level				
	Less than lower secondary education	19679 (42.08%)	4,233 (43.01%)	0.059
	Upper secondary and vocational training	17826 (38.12%)	3,757 (38.17%)	
	Tertiary education	9259 (19.8%)	1,852 (18.82%)	
Marital status				
	Married/partnered	34602 (73.99%)	7,131 (72.99%)	0.04
	Single	12162 (26.01%)	2,639 (27.01%)	
Smoke				
	Current smoker	8578 (18.34%)	1,823 (19.89%)	<0.001
	Non-current smoker	38186 (81.66%)	7,341 (80.11%)	
Drink				
	Yes	21065 (45.05%)	3,984 (43.55%)	0.009
	No	25699 (54.95%)	5,164 (56.45%)	
Labor force status				
	Working	12176 (26.04%)	2,362 (27.82%)	0.001
	Not working	34588 (73.96%)	6,128 (72.18%)	
Depressive symptoms				
	Yes	13794 (29.5%)	3,024 (30.73%)	0.015
	No	32970 (70.5%)	6,818 (69.27%)	
ADL impaired				
	Yes	5526 (11.82%)	1,459 (15.28%)	<0.001
	No	41238 (88.18%)	8,092 (84.72%)	
Presence of chronic illness				
	1 or more disease	27872 (59.6%)	5,557 (57.98%)	0.003
	None	18892 (40.4%)	4,028 (42.02%)	

Note: SHARE: Survey of Health, Ageing and Retirement in Europe; ADL: activities of daily living. Median (Q_{25}-Q_{75}) was used to describe continuous variables, and number (constituent ratio [%]) was used to describe categorical variables. The Kruskal-Wallis test for continuous variables and the Pearson chi-square test for categorical variables.

Table S14: Comparison summary of baseline characteristics between the included participants and excluded participants in CHARLS

**Characteristic**		**Included participants (n=11322)**	**Excluded participants (n=2319)**	*P*-value
Age		60 (56-67)	60 (55-70)	0.0765
Gender				
	Men	5548 (49%)	1225 (52.87%)	0.001
	Women	5774 (51%)	1092 (47.13%)	
Education level				
	Less than lower secondary education	10182 (89.93%)	1988 (86.32%)	<0.001
	Upper secondary and vocational training	961 (8.49%)	253 (10.99%)	
	Tertiary education	179 (1.58%)	62 (2.69%)	
Marital status				
	Married/partnered	9669 (85.4%)	1879 (81.38%)	<0.001
	Single	1653 (14.6%)	430 (18.62%)	
Smoke				
	Current smoker	3558 (31.43%)	344 (20%)	<0.001
	Non-current smoker	7764 (68.57%)	1376 (80%)	
Drink				
	Yes	4704 (41.55%)	949 (43.27%)	0.134
	No	6618 (58.45%)	1244 (56.73%)	
Labor force status				
	Working	6620 (58.47%)	1001 (50.92%)	<0.001
	Not working	4702 (41.53%)	965 (49.08%)	
Depressive symptoms				
	Yes	4401 (38.87%)	422 (38.57%)	0.847
	No	6921 (61.13%)	672 (61.43%)	
ADL impaired				
	Yes	2127 (18.79%)	486 (23.6%)	<0.001
	No	9195 (81.21%)	1573 (76.4%)	
Presence of chronic illness				
	1 or more disease	5116 (45.19%)	976 (47.36%)	0.069
	None	6206 (54.81%)	1085 (52.64%)	

Note: CHARLS: China Health and Retirement Longitudinal Study; ADL: activities of daily living. Median (Q_{25}-Q_{75}) was used to describe continuous variables, and number (constituent ratio [%]) was used to describe categorical variables. The Kruskal-Wallis test for continuous variables and the Pearson chi-square test for categorical variables.

Table S15. Frequency of patterns of change in hobby engagement across cohorts

Region/country	Frequency (%)			
	Sustained non-engagement	Initiation of engagement	Cessation of engagement	Sustained engagement
USA	4281 (33.83%)	1174 (9.28%)	2323 (18.35%)	4878 (38.54%)
England	2576 (13.44%)	1483 (7.74%)	2048 (10.69%)	13054 (68.13%)
China	11923 (65.11%)	1817 (9.92%)	1637 (8.94%)	2935 (16.03%)
Europe	4240 (9.5%)	3172 (7.1%)	3237 (7.25%)	34006 (76.15%)
Austria	118 (3.08%)	154 (4.03%)	127 (3.32%)	3426 (89.57%)
Belgium	164 (5.08%)	183 (5.67%)	213 (6.59%)	2670 (82.66%)
Czech Republic	163 (2.98%)	313 (5.72%)	321 (5.87%)	4676 (85.44%)
Denmark	21 (1.69%)	19 (1.53%)	26 (2.09%)	1178 (94.69%)
Estonia	362 (3.59%)	481 (4.77%)	628 (6.23%)	8606 (85.40%)
France	207 (6.23%)	265 (7.98%)	251 (7.56%)	2597 (78.22%)
Germany	24 (2.02%)	54 (4.54%)	39 (3.28%)	1072 (90.16%)
Italy	1,161 (33.26%)	441 (12.63%)	511 (14.64%)	1378 (39.47%)
Netherlands	11 (1.48%)	17 (2.29%)	31 (4.17%)	684 (92.06%)
Poland	127 (17.14%)	106 (14.30%)	83 (11.20%)	425 (57.35%)
Portugal	96 (22.07%)	48 (11.03%)	77 (17.70%)	214 (49.20%)
Slovenia	235 (8.16%)	360 (12.50%)	223 (7.74%)	2062 (71.60%)
Spain	1,493 (34.75%)	592 (13.78%)	579 (13.48%)	1632 (37.99%)
Sweden	8 (0.54%)	34 (2.31%)	37 (2.51%)	1394 (94.64%)
Switzerland	50 (2.23%)	105 (4.69%)	91 (4.07%)	1992 (89.01%)

Note: The change patterns of hobby engagement were categorized as (1) "*Sustained non-engagement*": No hobby engagement in either of the two exposure waves preceding the survival status assessment wave; (2) "*Initiation of engagement*": No hobby engagement in the first exposure wave, but hobby engagement in the second exposure wave; (3) "*Cessation of engagement*": Hobby engagement in the first exposure wave, but no hobby engagement in the second exposure wave; (4) "*Sustained engagement*": Hobby engagement in both exposure waves.

Table S16. Subgroup and interaction analysis

		HRS		ELSA		SHARE		CHARLS		Pooled	
		HR (95% CI)	*P*-value	HR (95% CI)	*P*-value	HR (95% CI)	*P*-value	HR (95% CI)	*P*-value	HR (95% CI)	*P* for heterogeneity
Age group											
	Interaction	0.97 (0.81-1.16)	0.716	0.75 (0.56-1.00)	0.053	0.77 (0.67-0.89)	<0.001	0.67 (0.49-0.90)	0.009	0.79 (0.68-0.91)	0.04
	50-64	0.82 (0.69-0.98)	0.032	0.86 (0.65-1.15)	0.306	0.82 (0.72-0.94)	0.006	0.88 (0.70-1.11)	0.291	0.84 (0.77-0.90)	0.94
	65 +	0.72 (0.67-0.77)	<0.001	0.60 (0.53-0.68)	<0.001	0.62 (0.58-0.66)	<0.001	0.61 (0.50-0.75)	<0.001	0.64 (0.58-0.70)	<0.001
Gender											
	Interaction	0.92 (0.82-1.05)	0.216	1.12 (0.90-1.39)	0.298	1.08 (0.98-1.19)	0.109	1.18 (1.85-1.66)	0.326	1.03 (0.92-1.16)	0.16
	Men	0.78 (0.71-0.86)	<0.001	0.73 (0.63-0.86)	<0.001	0.74 (0.69-0.80)	<0.001	0.75 (7.66-0.90)	0.002	0.75 (0.72-0.79)	0.72
	Women	0.82 (0.75-0.90)	<0.001	0.67 (0.57-0.78)	<0.001	0.67 (0.62-0.73)	<0.001	0.64 (6.66-0.86)	0.003	0.70 (0.63-0.79)	<0.001
Education level											
	Upper secondary and vocational	1.00 (0.85-1.16)	0.974	0.81 (0.64-1.04)	0.097	0.78 (1.69-0.88)	<0.001	1.10 (0.59-2.02)	0.768	0.86 (0.75-1.00)	0.06

	training * hobby										
	Tertiary education * hobby	0.92 (0.75-1.13)	0.411	1.47 (0.82-2.65)	0.195	0.80 (0.64-1.00)	0.048	0.18 (0.04-0.81)	0.026	0.64 (0.26-1.56)	<0.001
	Less than lower secondary education	0.79 (0.69-0.91)	0.001	0.72 (0.63-0.83)	<0.001	0.74 (0.69-0.79)	<0.001	0.73 (0.62-0.85)	<0.001	0.74 (0.72-0.77)	0.62
	Upper secondary and vocational training	0.81 (0.75-0.88)	<0.001	0.63 (0.51-0.78)	<0.001	0.59 (0.53-0.66)	<0.001	0.89 (0.48-1.63)	0.699	0.69 (0.57-0.83)	<0.001
	Tertiary education	0.81 (0.69-0.95)	0.011	1.10 (0.62-1.97)	0.737	0.61 (0.49-0.77)	<0.001	0.15 (0.03-0.76)	0.022	0.52 (0.22-1.25)	<0.001
Marital status											
	Interaction	0.89 (0.79-1.01)	0.078	1.05 (0.84-1.30)	0.684	0.89 (0.80-0.98)	0.019	1.28 (0.88-1.86)	0.198	0.91 (0.85-0.97)	0.28
	Married/partnered	0.79 (0.72-0.86)	<0.001	0.72 (0.62-0.84)	<0.001	0.69 (0.65-0.74)	<0.001	0.75 (0.63-0.89)	0.001	0.74 (0.69-0.79)	0.03
	Single	0.83 (0.76-0.92)	<0.001	0.68 (0.58-0.81)	<0.001	0.72 (0.66-0.79)	<0.001	0.60 (0.43-0.85)	0.003	0.72 (0.64-0.81)	0.002
Labor force status											

	Interaction	0.95 (0.81-1.12)	0.564	1.36 (0.89-2.09)	0.159	1.27 (0.94-1.71)	0.12	1.37 (1.01-1.86)	0.046	1.14 (0.91-1.42)	0.20
	Working	0.82 (0.70-0.96)	0.013	1.05 (0.68-1.61)	0.826	0.97 (0.71-1.33)	0.859	0.84 (0.66-1.07)	0.158	0.85 (0.77-0.94)	0.60
	Not working	0.79 (0.74-0.85)	<0.001	0.68 (0.61-0.77)	<0.001	0.69 (0.65-0.73)	<0.001	0.67 (0.55-0.81)	<0.001	0.71 (0.66-0.77)	<0.001
Depressive symptoms											
	Interaction	1.18 (1.02-1.36)	0.022	1.23 (0.98-1.55)	0.07	1.02 (1.92-1.13)	0.689	0.78 (0.56-1.08)	0.136	1.04 (0.88-1.24)	0.03
	Yes	0.87 (0.77-0.99)	0.035	0.78 (0.65-0.95)	0.011	0.68 (0.63-0.73)	<0.001	0.60 (0.45-0.79)	<0.001	0.73 (0.62-0.85)	<0.001
	No	0.78 (0.73-0.84)	<0.001	0.67 (0.58-0.77)	<0.001	0.71 (0.66-0.77)	<0.001	0.79 (0.65-0.95)	0.012	0.73 (0.68-0.79)	0.01
ADL impaired											
	Interaction	1.00 (0.87-1.14)	0.949	0.96 (0.77-1.20)	0.736	0.90 (1.81-1.00)	0.051	1.19 (0.85-1.68)	0.307	0.95 (0.87-1.02)	0.43
	Yes	0.77 (0.68-0.87)	<0.001	0.66 (0.55-0.79)	<0.001	0.63 (0.57-0.69)	<0.001	0.83 (0.61-1.12)	0.214	0.70 (0.62-0.79)	0.001
	No	0.82 (0.76-0.89)	<0.001	0.74 (0.64-0.85)	<0.001	0.75 (0.70-0.80)	<0.001	0.69 (0.58-0.82)	<0.001	0.76 (0.71-0.81)	0.03

Note: HRS: Health and Retirement Study; ELSA: English Longitudinal Study of Ageing; SHARE: Survey of Health, Ageing and Retirement in Europe; CHARLS: China Health and Retirement Longitudinal Study; HR: Hazard ratio; CI: Confidence Interval; ADL: activities of daily living.

Table S17. Subgroup analyses by hobbies measures in meta-analysis

Hobbies measures	HR	95% CI	I^2	H^2	$P_{subgroup}$
Listed	0.693	0.656-0.732	5.18%	1.05	0.193
Binary	0.758	0.670-0.858	73.32%	3.75	

Note: N study = 19. Hobbies in Europe (SHARE) and China (CHARLS) were measured with listed, whereas hobbies in the USA (HRS) and England (ELSA) were measured with binary. I^2 indicates the percentage of variability in the effect size that is caused by between-study heterogeneity, rather than by sampling error. A value of I^2>50% indicates heterogeneity. H^2 statistics describe the ratio of the observed variation and the expected variance due to sampling error. A value of H^2greater than 1 indicates the presence of between-study heterogeneity. $P_{subgroup}$ assesses whether the effect sizes differ significantly between predefined subgroups within the meta-analysis.

Table S18. The E-value of association of hobby engagement and mortality.

Cohort	E-value [a]	CI limit
HRS	1.606	1.474
ELSA	1.864	1.637
SHARE	1.867	1.754
CHARLS	1.834	1.524

Note: HRS, Health and Retirement Study; CHARLS, China Health and Retirement Longitudinal Study; SHARE, Survey of Health, Ageing and Retirement in Europe; ELSA, English Longitudinal Study of Ageing.

[a] Model was adjusted for age, gender, education level, marital status, smoke, drink, labor force status, depressive symptoms, ADL impaired, presence of chronic illness.

Table S19. Cox proportional analyses with multiple imputations

Cohort	N	HR	95% CI	*P* value
HRS	13,708	0.80	0.75-0.85	<0.001
ELSA	9,031	0.72	0.65-0.80	<0.001
SHARE	47411	0.71	0.67-0.75	<0.001
CHARLS	11951	0.70	0.61-0.82	<0.001

Note: HRS, Health and Retirement Study; CHARLS, China Health and Retirement Longitudinal Study; SHARE, Survey of Health, Ageing and Retirement in Europe; ELSA, English Longitudinal Study of Ageing.

Table S20. Cox proportional analyses with survey weight

Cohort	N	HR	95% CI	*P* value
HRS	12,871	0.82	0.76-0.89	<0.001
ELSA	8,053	0.71	0.64-0.79	<0.001
SHARE	46,605	0.69	0.61-0.77	<0.001
CHARLS	11,322	0.76	0.59-0.97	0.025

Note: HRS, Health and Retirement Study; CHARLS, China Health and Retirement Longitudinal Study; SHARE, Survey of Health, Ageing and Retirement in Europe; ELSA, English Longitudinal Study of Ageing.

Table S21. Cox proportional analyses excluding respondents with chronic illness (cancer, heart disease, stroke, diabetes, lung disease, or hypertension) at baseline

Cohort	N	HR	95% CI	*P* value
HRS	3,506	0.77	0.64-0.93	0.006
ELSA	3,946	0.69	0.56-0.86	0.001
SHARE	18,892	0.71	0.63-0.80	<0.001
CHARLS	6,206	0.76	0.60-0.98	0.032

Note: HRS, Health and Retirement Study; CHARLS, China Health and Retirement Longitudinal Study; SHARE, Survey of Health, Ageing and Retirement in Europe; ELSA, English Longitudinal Study of Ageing.

Table S22. Cox proportional analyses with further inclusion of total household wealth

Cohort	N	HR	95% CI	*P* value
HRS	13,235	0.81	0.76-0.86	<0.001
ELSA	8,022	0.72	0.64-0.81	<0.001
SHARE	46,764	0.70	0.67-0.74	<0.001
CHARLS	7,770	0.70	0.59-0.84	<0.001

Note: HRS, Health and Retirement Study; CHARLS, China Health and Retirement Longitudinal Study; SHARE, Survey of Health, Ageing and Retirement in Europe; ELSA, English Longitudinal Study of Ageing.

Total household wealth was measured as the sum of all wealth components (including residence, vehicles, saving accounts, etc.) minus other debts at the couple level (the respondent and spouse, if any) in local currencies, further categorized into high, medium, and low groups based on tertile divisions [18].

Table S23. Meta-analysis of the association between hobby engagement and all-cause mortality risk across cohort studies (except the ELSA)

Cohort	HR	95% CI	% Weight
HRS	0.64	0.59-0.70	31.64
SHARE	0.70	0.68-0.73	53.37
CHARLS	0.72	0.62-0.84	14.99
Overall	0.69	0.64-0.74	100.00

Note: HRS, Health and Retirement Study; CHARLS, China Health and Retirement Longitudinal Study; SHARE, Survey of Health, Ageing and Retirement in Europe. I^2=50.96%, H^2=2.04, *P* for heterogeneity = 0.13.

Reference

1 Sonnega A, Faul JD, Ofstedal MB, Langa KM, Phillips JW, Weir DR. Cohort Profile: the Health and Retirement Study (HRS). International Journal of Epidemiology. 2014;43:576–85.

2 Steven G H, Judith H. C. Technical Description of the Health and Retirement Survey Sample Design. Sampling Section Institute for Social Research University of Michigan Ann Arbor, MI; 1995. Available: https://hrsonline.isr.umich.edu/sitedocs/userg/HRSSAMP.pdf

3 Mak HW, Noguchi T, Bone JK, Wels J, Gao Q, Kondo K, et al. Hobby engagement and mental wellbeing among people aged 65 years and older in 16 countries. Nat Med. 2023;29:2233–40.

4 Cheshire H, Ofstedal MB, Scholes S, Schroeder M. A comparison of response rates in the English Longitudinal Study of Ageing and the Health and Retirement Study. Longitudinal and life course studies. 2011;2:127.

5 Weir DR. Validating mortality ascertainment in the health and retirement study. 2016. Available: https://hrs.isr.umich.edu/sites/default/files/biblio/Weir_mortality_ascertainment.pdf

6 Alimujiang A, Wiensch A, Boss J, Fleischer NL, Mondul AM, McLean K, et al. Association Between Life Purpose and Mortality Among US Adults Older Than 50 Years. JAMA Network Open. 2019;2:e194270.

7 Gao Q, Bone JK, Bu F, Paul E, Sonke JK, Fancourt D. Associations of Social, Cultural, and Community Engagement With Health Care Utilization in the US Health and Retirement Study. JAMA Network Open. 2023;6:e236636.

8 Steptoe A, Breeze E, Banks J, Nazroo J. Cohort Profile: The English Longitudinal Study of Ageing. International Journal of Epidemiology. 2012;42:1640.

9 Börsch-Supan A, Brandt M, Hunkler C, Kneip T, Korbmacher J, Malter F, et al. Data Resource Profile: The Survey of Health, Ageing and Retirement in Europe (SHARE). International Journal of Epidemiology. 2013;42:992–1001.

10 Michael B, Thorsten K, Giuseppe DL, Annette S. Survey participation in the Survey of Health, Ageing and Retirement in Europe (SHARE), Wave 1-7. 2019. Available: https://share-eric.eu/fileadmin/user_upload/SHARE_Working_Paper/WP_Series_41_2019_Bergmann_et_al.pdf

11 Yuan Y, Si H, Shi Z, Wang Y, Xia Y, Guan X, et al. Association of Cognitive Frailty With Subsequent All-Cause Mortality Among Middle-Aged and Older Adults in 17 Countries. The American Journal of Geriatric Psychiatry. 2024;33:178–91.

12 Addington-Hall J, McPherson C. After-Death Interviews with Surrogates/Bereaved Family Members. Journal of Pain and Symptom Management. 2001;22:784–90.

13 Zhao Y, Strauss J, Yang G, Giles J, Hu P, Hu Y, et al. China health and retirement longitudinal study–2011–2012 national baseline users' guide. Beijing: National School of Development,

Peking University. 2013;2.

14 Zhao Y, Hu Y, Smith JP, Strauss J, Yang G. Cohort profile: the China health and retirement longitudinal study (CHARLS). International journal of epidemiology. 2014;43:61–8.

15 Gilsanz P, Walter S, Tchetgen Tchetgen EJ, Patton KK, Moon JR, Capistrant BD, et al. Changes in Depressive Symptoms and Incidence of First Stroke Among Middle-Aged and Older US Adults. Journal of the American Heart Association. 2015;4:e001923.

16 Mahmood A, Ray M, Ward KD, Dobalian A, Ahn S. Longitudinal associations between insomnia symptoms and all-cause mortality among middle-aged and older adults: a population-based cohort study. Sleep. 2022;45:zsac019.

17 Robins JM. Association, Causation, and Marginal Structural Models. Synthese. 1999;121:151–79. Available: https://www.jstor.org/stable/20118224

18 Pool LR, Burgard SA, Needham BL, Elliott MR, Langa KM, Mendes de Leon CF. Association of a Negative Wealth Shock With All-Cause Mortality in Middle-aged and Older Adults in the United States. JAMA. 2018;319:1341–50.

19 Cole SR, Hernán MA. Constructing inverse probability weights for marginal structural models. Am J Epidemiol. 2008;168:656–64.

20 Suarez D, Haro JM, Novick D, Ochoa S. Marginal structural models might overcome confounding when analyzing multiple treatment effects in observational studies. J Clin Epidemiol. 2008;61:525–30.

21 Daskalopoulou C, Prince M, Koukounari A, Haro JM, Panagiotakos DB, Prina AM. Healthy ageing and the prediction of mortality and incidence dependence in low- and middle- income countries: a 10/66 population-based cohort study. BMC Med Res Methodol. 2019;19:225.

22 Li S, Stampfer MJ, Williams DR, VanderWeele TJ. Association of Religious Service Attendance With Mortality Among Women. JAMA Internal Medicine. 2016;176:777–85.

23 After-Death Interviews with Surrogates/Bereaved Family Members: Some Issues of Validity. Journal of Pain and Symptom Management. 2001;22:784–90.

24 Gao J, Qiu Y, Hou Y, Zhang L, Wang K, Chen Z, et al. Influencing factors for the decline of limb muscle strength and the association with all-cause mortality: evidence from a nationwide population-based cohort study. Aging Clin Exp Res. 2022;34:399–407.

25 Guo Y, Qian D. Workforce Participation and Mortality Risk Among Chinese Older Adults: A Nationwide Population-Based Prospective Study. Schafer M, editor. The Journals of Gerontology: Series B. 2023;78:1947–56.

26 Wang D, Dai X, Mishra SR, Lim CCW, Carrillo-Larco RM, Gakidou E, et al. Association between socioeconomic status and health behaviour change before and after non-

communicable disease diagnoses: a multicohort study. The Lancet Public Health. 2022;7:e670–82.

27 Lu X, Yao Y, Jin Y. Digital exclusion and functional dependence in older people: Findings from five longitudinal cohort studies. eClinicalMedicine. 2022;54.
